# Rest–Activity Timing Phenotypes and Mental Health: A Longitudinal Analysis of 24-Hour Accelerometry in Population-Based Cohorts

**DOI:** 10.34133/hds.0306

**Published:** 2026-03-03

**Authors:** Yanbo Wang, Lihui Zhou, Mengjing Zhu, Hongxi Yang, Dun Li, Jing Lin, Yanchun Chen, Qi Lu, Li Sun, Xinyu Zhang, Yue Zhao, Wenli Lu, Chenjie Xu, Yaogang Wang

**Affiliations:** ^1^ School of Public Health, Tianjin Medical University, Tianjin, China.; ^2^ Department of Bioinformatics, School of Basic Medical Sciences, Tianjin Medical University, Tianjin, China.; ^3^ School of Nursing, Tianjin Medical University, Tianjin, China.; ^4^ School of Public Health, Hangzhou Normal University, Hangzhou, China.; ^5^ School of Integrative Medicine, Public Health Science and Engineering College, Tianjin University of Traditional Chinese Medicine, Tianjin, China.; ^6^ National Institute of Health Data Science at Peking University, Peking University, Beijing, China.

## Abstract

**Background:** Questionnaire-based chronotype measures may be affected by reporting bias. In contrast, 24-h accelerometry objectively captures rest–activity timing phenotypes as a behavioral proxy for chronotype. We examined whether accelerometer-derived activity timing phenotypes are linked to the onset of major depressive disorder (MDD) and anxiety disorders. **Methods:** With the UK Biobank, 94,344 participants free of MDD and anxiety disorders with valid accelerometry data were included. Accelerometer-derived rest–activity timing phenotypes were classified via *k*-means clustering of 24-h activity patterns. Cox models calculated hazard ratios (HRs) for the onset of MDD and anxiety. Brain magnetic resonance imaging (MRI) biomarkers were analyzed in 17,571 participants. **Results:** A total of 2,361 MDD and 2,278 anxiety cases were identified over a median 6.8-year follow-up period. Compared with participants in the accelerometer-derived daytime-active group, the night-active group had higher risks of MDD [1.30; 95% confidence interval (CI): 1.15 to 1.48] and anxiety disorders (1.27; 95% CI: 1.11 to 1.45). The early-morning-active phenotype group showed a 19% (95% CI: 8% to 29%) reduced risk of MDD. High physical activity (PA) levels in the period of 00:00 to 04:59 showed adverse effects for MDD and anxiety disorders, while high PA levels in the period of 06:00 to 08:59 showed favorable effects. In addition, the night-active group was linked to lower volume of white matter and gray matter, lower frontoparietal gray matter volumes, and lower subcortical volumes. **Conclusions:** Objectively measured activity timing phenotypes were associated with incident MDD and anxiety, and showed exploratory MRI correlates that may help generate mechanistic hypotheses. Whether modifying activity timing reduces mental disorder risk requires randomized controlled trials.

## Introduction

Numerous physiological functions fluctuate across the 24-h day, and the circadian system plays an essential role in regulating energy metabolism, immune-related processes, as well as cellular repair and renewal [1,2]. Timing profiles for exercise, sleep–wake cycles, and other behaviors might regulate immune function, monoamine transmission, neurogenesis, and metabolism, which further alter mental health [3,4]. Accumulating evidence indicates that higher levels of physical activity (PA) are linked to reduced risks of depression and anxiety [5]. However, beyond PA intensity and cumulative amount, the timing pattern of PA across the 24-h day (i.e., rest–activity timing phenotypes derived from wearables) may be associated with mental health outcomes [6,7].

Previous studies used self-reported chronotype to reflect individuals’ preference for timing activities across the day and sleep–wake schedules. Studies have suggested that self-reported evening chronotype was linked to higher levels of depression and accelerated brain aging [8]. Nevertheless, several studies have reported the discrepancies between self-reported and objectively assessed PA information, which might even distort effect sizes for health outcomes [9,10]. In addition, self-reported chronotypes might not accurately reflect chronotypes partly due to reporting bias [11]. Thus, objective and scalable measures of free-living rest–activity timing are needed to complement questionnaire-based chronotype assessments. Wearable devices, such as accelerometers, can continuously monitor behaviors and provide objective measures of 24-h rest–activity timing phenotypes, enabling the derivation of behavioral timing profiles in free-living settings (hereafter referred to as accelerometer-derived rest–activity timing phenotypes) [12–14]. According to a cross-sectional analysis of the Look AHEAD cohort, men who were most active in the morning hours measured by a wrist-worn accelerometer tended to exhibit the greatest cardiorespiratory fitness, whereas those whose moderate-to-vigorous PA (MVPA) peaked during daytime showed the lowest fitness levels [6]. Nikbakhtian et al. have revealed that accelerometer-derived “early morning peak” and “late morning peak” chrono-activity types were associated with lower cardiovascular disease risk relative to an average PA timing pattern [15]. However, the associations of measured behavioral chronotype with mental health remain unclear.

Importantly, the biological pathways linking chronotype to mental health are still not fully understood. Disruption of circadian rhythms may disturb hypothalamic–pituitary–adrenal (HPA) axis regulation, resulting in abnormal cortisol secretion patterns that have been consistently linked to depression and anxiety [16,17]. Additionally, circadian disruption can affect monoaminergic neurotransmission, particularly serotonergic and noradrenergic signaling, which are strongly implicated in mood regulation [4,17]. Dopaminergic pathways may also be involved, although their contribution appears more context-dependent and may be particularly relevant in late-life affective symptoms or substance use disorders [18–20]. Neuroinflammatory processes, including microglial activation and cytokine release, are also under circadian control, and their dysregulation may contribute to neuronal damage and psychiatric symptoms [16,21]. These molecular and cellular disturbances may manifest as structural alterations in the brain. Furthermore, chronic circadian disturbance and sleep irregularity may influence neuroplasticity-related processes (including synaptic remodeling and myelin integrity), potentially manifesting as differences in brain macrostructural measures and microstructural properties captured by structural and diffusion magnetic resonance imaging (MRI) [17,22]. Brain structure is often treated as an intermediate phenotype that helps explain vulnerability to mental disorders [12,23], as it reflects both genetic influences and neurobiological pathology. Accordingly, chronotype-related differences in brain structure and function may contribute directly to mental health problems [12]. Therefore, investigating the associations between accelerometer-derived rest–activity timing phenotypes and MRI-based brain structural features may provide mechanistic insights into how behavioral chronotypes influence mental health outcomes.

With data from one of the largest prospective cohorts, the UK Biobank, the current study aimed to examine the associations of accelerometer-derived rest–activity timing phenotypes with the incidence risk of major depressive disorder (MDD) and anxiety disorders. We also investigated the associations of accelerometer-derived PA intensity of each hour over the 24-h cycle with MDD and anxiety disorders to provide insights and complementary understanding into the associations of activity time patterns with mental health outcomes. Further, this study also examined the associations of accelerometer-derived rest–activity timing phenotypes with MRI-based brain structural features.

## Methods

### Study design and population

Our primary analyses comprised prospective cohort analyses of incident MDD and anxiety disorders in relation to accelerometer-derived rest–activity timing phenotypes and hourly PA intensity. Baseline was defined as the date of the accelerometer assessment, when rest–activity timing phenotypes were derived. Participants with the outcomes of interest at or before baseline were excluded, and the remaining participants were followed from baseline to the first outcome event, death, or follow-up end. In addition, we conducted secondary (exploratory) neuroimaging analyses in the subset of participants with available MRI data; details are described in the Statistical analysis section.

This study used UK Biobank data, a large open-access cohort that enrolled approximately 500,000 participants aged 37 to 73 years from 22 assessment centers across England, Wales, and Scotland. This study was implemented under approval number 45676. The cohort design and procedures have been reported previously [24]. Briefly, baseline characteristics and physical examinations were collected at assessment centers from 2006 to 2010. Participants were followed using linked hospital admission records and death registers.

From May 2013 to December 2015, a randomly selected subset of participants with valid email addresses was invited to complete 7 consecutive days of PA monitoring [25]. A total of 103,683 participants completed tests using the Axivity AX3 wrist-worn triaxial device, which is the commercial implementation of the Open Movement AX3 open-source sensor (https://github.com/digitalinteraction/openmovement).

In this analysis, 6,999 participants with <3 d of wear, missing data in every 1-h interval of the 24-h cycle, implausible average acceleration [>500 milligravity (mg)], or poor calibration were excluded. Further, 2,340 participants were excluded due to prevalent outcomes of interest at baseline, and 23 participants were excluded because of the lack of key information (age and gender). In total, 94,344 participants were included (Fig. S1), representing a retention rate of approximately~91% among those who completed the accelerometer assessment.

### Accelerometer-derived rest–activity timing phenotypes and measured PA intensity of each hour

The Axivity device recorded 7 d of continuous triaxial acceleration at 100 Hz (dynamic range: ±8*g*). To ensure consistency across devices, the acceleration signals were adjusted with reference to the local gravitational value [25,26]. The distribution of time spent on different PA intensity levels was characterized using an empirical cumulative distribution function in 5-s epochs from the combination of the sample-level data [25,27]. Accelerometer-measured PA intensity was expressed in milligravity units (mg), where 1 mg equals 0.00981 m/s^2^ [25,27].

The relative average acceleration of each hour of the 24-h cycle was derived by dividing the hourly average acceleration by the no-wear time bias-adjusted average acceleration. Accelerometer-derived rest–activity timing phenotypes were defined using *k*-means clustering analysis on *Z*-score transformed hourly relative average accelerations. Using this approach, we identified 4 accelerometer-derived rest–activity timing phenotypes based on 24-h activity profiles. Participants were assigned to clusters based on the similarity of their entire 24-h rest–activity profiles (24-dimensional vectors of *Z*-scored hourly acceleration), rather than solely on the timing of peak activity. Accordingly, the phenotypes were labeled descriptively according to the period with relatively higher activity: early-morning-active (06:00 to 07:59), daytime-active (08:00 to 19:59), evening-active (20:00 to 00:59), and night-active (01:00 to 05:59).

In this study, accelerometer-derived rest–activity timing phenotypes are interpreted as behavioral timing patterns that may reflect a mixture of endogenous circadian regulation and exogenous constraints (e.g., work schedules, habits, and light exposure), rather than direct measures of endogenous circadian phase or rhythm amplitude [28].

**Fig. 1.**
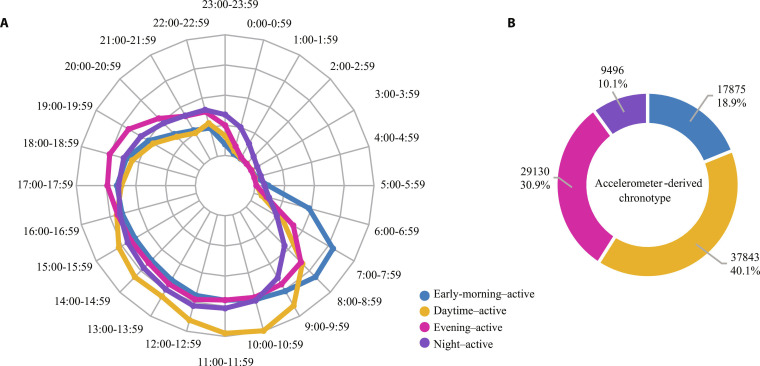
The 24-h relative average accelerations of the 4 accelerometer-derived rest–activity timing phenotypes using *k*-means clustering analysis and proportion of accelerometer-derived rest–activity timing phenotypes. (A) The 24-h relative average accelerations of the 4 accelerometer-derived rest–activity timing phenotypes. (B) Number and proportion of participants in the 4 accelerometer-derived rest–activity timing phenotypes.

To avoid the effect of outliers, the average accelerations of each hour were winsorized at 2.5th and 97.5th. The “elbow” method was applied to ascertain the best number of clusters [29]. The within-groups sums of squares (WSSs) of 1 to 15 clusters were calculated, and the optimal cluster number was the elbow position of the “elbow” chart (Fig. S2). The 24-h relative average acceleration profiles of the 4 rest–activity timing phenotypes (cluster centers) are shown in Fig. 1A. The early-morning-active phenotype had higher relative accelerations in the 06:00 to 07:59 period among all 4 types (*n* = 17,875). There were 37,843 (40.1%) participants in the daytime-active phenotype, and they had the highest relative acceleration in 08:00 to 19:59 among all 4 types. The evening-active (*n* = 29,130) and night-active (*n* = 9,496) phenotypes had higher relative accelerations in 20:00 to 00:59 and 01:00 to 05:59, respectively, among all 4 types. The daytime-active phenotype was used as the reference in the analysis (Fig. 1B).

Further, we investigated the associations of the activity intensity of every 1-h interval of the 24-h cycle with MDD and anxiety disorders. The hazard ratios (HRs) of hourly activity intensity with MDD and anxiety disorders were analyzed by comparing participants with high PA levels (above the median) and those with low PA levels (at or below the median). The description of hourly PA intensity was listed in Table S1.

### Outcomes defined

The primary outcomes were the incidence of MDD and anxiety disorders, defined by International Classification of Diseases, 10th Revision (ICD-10) codes F32/F33 and F40/F41, respectively. Hospital admission dates and diagnoses were ascertained through linkage to Health Episode Statistics (England and Wales), the Scottish Morbidity Records (Scotland), and the death registry.

Secondary outcomes included MRI-derived indices relevant to mental health. Imaging was acquired on a Siemens Skyra 3T system (VD13A SP4) using a standard Siemens 32-channel radio frequency head coil. UK Biobank documentation specifies the imaging protocol (https://biobank.ndph.ox.ac.uk/ukb/ukb/docs/brain_mri.pdf). Imaging data underwent automated processing and quality control using an openly available pipeline [30]. We analyzed a selected subset of imaging-derived phenotypes in the present study. The indicators comprised total brain volume, white matter hyperintensity (WMH), white matter (WM) volume, gray matter (GM) volume, tract-averaged fractional anisotropy (FA), and mean diffusivity (MD) for WM tracts listed in Table S3, as well as subcortical volumes. These biomarkers have previously been implicated in mental health [23,31]. We excluded extreme values located more than ±4 standard deviations from the mean on a case-wise basis.

### Covariates

We have identified 11 covariates as potential confounders of the association between rest–activity timing and mental health outcomes , based on prior literature [32–34]. First, the sociodemographic information included age at the accelerometry test, sex, ethnicity (white or others), education levels (college or university degree, A levels/AS levels or equivalent, O levels/GCSEs or equivalent, or other qualifications), and quintiles of the Townsend deprivation index (TDI). Second, lifestyle behaviors included smoking status (current, previous, or never), alcohol drinking frequency (daily/nearly daily, 3 to 4 times/week, 1 to 2 times/week, 1 to 3 times/month, special occasions only, never), diet quality score (low [0 to 1], medium [2, 3], and high [4, 5]), body mass index (BMI), sleep quality score (low [0 to 1], medium [2, 3], and high [4, 5]), and accelerometer-derived minutes per week of MVPA (defined using the 125-mg cutoff) [35]. The diet quality score was derived from prior work and reflected adherence to key Mediterranean diet components, including higher intakes of vegetables, fruit, and fish and lower intakes of unprocessed red and processed meats. The sleep quality score was calculated using 5 sleep-related, self-reported items. Good sleep quality was defined by no snoring, no frequent daytime sleepiness, normal sleep duration, getting up easily in the morning, and reported never or rarely insomnia [36]. Higher scores indicated higher diet or sleep qualities. Data sources and covariate definitions are summarized in Table S2.

### Statistical analysis

Participant characteristics overall and by accelerometer-derived rest–activity timing phenotype were summarized as mean (SD) or median [interquartile range (IQR)] for continuous variables and as number (percentage) for categorical variables.

The follow-up duration started at the date of the accelerometry assessment (conducted between May 2013 and December 2015) and ended with the first date of hospitalization for outcomes of interest or the end of follow-up (England: 30 September 2021; Scotland: 21 September 2021; Wales: 31 May 2016), whichever came first. We applied Cox proportional hazards models to estimate HRs with 95% confidence intervals (CIs) of accelerometer-derived rest–activity timing phenotypes (daytime-active as the reference) and PA intensity level of each 1-h interval of the 24-h cycle for MDD and anxiety disorders, adjusted for the aforementioned covariates. The proportional hazards assumption was evaluated by visually inspecting Schoenfeld residuals. No multicollinearity was identified [maximum generalized variance inflation factor (VIF): 1.32]. In the main analyses, missing covariate values were handled using multiple imputation by chained equations with 5 imputations. Bonferroni adjustment was applied to account for multiple comparisons within each analysis.

In the subgroup analysis, the modification effects of age groups (<60 years and ≥60 years), gender (males and females), and levels of measured MVPA [high and low PA levels defined using the median (470 min/week) as the cutoff point] on the associations between rest–activity timing phenotypes and hourly PA intensity level with the outcomes were examined. *P* for multiplicative interactions was calculated.

To evaluate robustness and potential confounding, we conducted a series of supplementary analyses (Figs. S6 to S16). We (a) re-ran the analyses among participants with complete covariate data (complete-case analysis); (b) performed 1- and 2-year landmark analyses by excluding participants who developed events of interest within 1 or 2 years after the accelerometer assessment to reduce potential reverse causation; (c) used *E* values to quantify how unmeasured confounding could affect the associations between accelerometer-derived rest–activity timing phenotypes and mental health outcomes [*E* values represent the minimum strength of association (on the risk ratio scale) required to fully explain away an observed association. Specifically, this is the strength that an unmeasured confounder would need to have with both the accelerometer-derived rest–activity timing phenotypes and the outcome, conditional on the measured covariates. Therefore, larger *E* values indicate more robust associations [37]; (d) excluded participants with baseline psychotic disorders, bipolar disorder, attention-deficit hyperactivity disorder, or substance use disorders; (e) restricted analyses to those not reporting current shift or night shift work at baseline and without baseline sleep disorders; (f) fitted extended models additionally adjusting for prevalent hypertension, diabetes, cancer, and sleep disorders; and (g) assessed potential selection related to neuroimaging participation by comparing characteristics of participants with versus without MRI data (Table S4) using appropriate tests for continuous and categorical variables and quantifying between-group imbalance with standardized mean differences (SMDs; see Table S4 for details), and repeating the main analyses in the MRI subcohort. Ascertainment of comorbidities and shift work status is detailed in Table S6.

In secondary (exploratory) analyses, we examined cross-sectional associations between accelerometer-derived rest–activity timing phenotypes and neuroimaging measures in the subset of participants with available MRI data to provide mechanistic clues. We used multiple linear regression to explore the links between accelerometer-derived rest–activity timing phenotypes and MRI indices, adjusted for potential confounders. Multicollinearity was assessed using VIFs. *P* values were adjusted for multiple testing using the false discovery rate (FDR) control [38]. WMH values were log-transformed (offset = 1) to improve normality, and MRI indices were standardized as *Z* scores. To capture shared variance in WM microstructure across the brain, we used principal component analysis to derive the first principal components for fractional anisotropy (gFA) and mean diffusivity (gMD). Tract loadings and model fit were shown in Table S3.

Analyses were performed in R (version 4.4.1; R Foundation for Statistical Computing). All tests were 2-sided, with *α* = 0.05.

## Results

### Population characteristics

After applying exclusion criteria, 94,344 participants were included in the prospective analyses, of whom 17,571 had available neuroimaging data for cross-sectional MRI analyses (Fig. S1). The analysis sample comprised 41,459 (43.9%) males, and the mean age was 62.32 (±7.9) years. The characteristics of participants by 4 accelerometer-derived rest–activity timing phenotypes are listed in Table 1. Among all, participants in the accelerometer-derived night-active phenotype tended to be male, non-white, and have lower educational attainment and higher deprivation levels. They were also more likely to be current or previous smokers and have lower alcohol drinking frequency, lower diet quality score, higher sleep quality score, and higher BMI. Compared with participants without MRI, the imaging subset was slightly younger and had a modestly more advantaged socioeconomic/health profile (most SMDs <0.10; maximum approximately 0.11; Table S4).

**Table 1.** Characteristics of participants according to accelerometer-derived rest–activity timing phenotypes

Characteristics	All participants (*n* = 94,344)	Early-morning-active (*n* = 17,875)	Daytime-active (*n* = 37,843)	Evening-active (*n* = 29,130)	Night-active (*n* = 9,496)
Age at accelerometry test, years [mean (SD)]	62.32 (7.9)	59.88 (7.9)	65.07 (6.7)	59.92 (7.9)	63.34 (7.9)
Male [no. (%)]	41,459 (43.9)	8,640 (48.3)	16,429 (43.4)	11,355 (39.0)	5,035 (53.0)
White [no. (%)]	91,102 (96.9)	17,070 (95.8)	37,213 (98.7)	27,937 (96.2)	8,882 (94.0)
College or university degree [no. (%)]	40,735 (47.5)	7,564 (46.1)	14,152 (42.7)	15,158 (54.8)	3,861 (45.8)
Townsend deprivation index at recruitment (median [IQR])	−2.45 [−3.82, −0.20]	−2.25 [−3.69, 0.14]	−2.75 [−3.96, −0.90]	−2.37 [−3.79, −0.01]	−1.63 [−3.42, 1.23]
Smoking status [no. (%)]					
Never	53,920 (57.3)	10,437 (58.5)	21,272 (56.4)	17,639 (60.7)	4,572 (48.3)
Previous	33,793 (35.9)	6,238 (35.0)	14,378 (38.1)	9,594 (33.0)	3,583 (37.9)
Current	6,383 (6.8)	1,152 (6.5)	2,084 (5.5)	1,837 (6.3)	1,310 (13.8)
Alcohol drinking frequency [no. (%)]					
At least 3 times a week	46,297 (49.1)	8,014 (44.9)	19,680 (52.0)	14,563 (50.0)	4,040 (42.6)
Less than 3 times a week	33,949 (36.0)	6,727 (37.7)	13,260 (35.1)	10,573 (36.3)	3,389 (35.7)
Special occasions only or never	14,023 (14.9)	3,123 (17.5)	4,875 (12.9)	3,968 (13.6)	2,057 (21.7)
Diet quality score [no. (%)]					
Low (0–1)	9,443 (10.0)	1,878 (10.5)	3,391 (9.0)	2,861 (9.8)	1,313 (13.8)
Medium (2–3)	48,695 (51.6)	9,066 (50.7)	19,700 (52.1)	14,770 (50.7)	5,159 (54.3)
High (4–5)	36,206 (38.4)	6,931 (38.8)	14,752 (39.0)	11,499 (39.5)	3,024 (31.8)
Sleep quality score [no. (%)]					
Low (0–1)	43,780 (49.5)	8,629 (51.6)	17,907 (50.1)	14,027 (51.2)	3,217 (37.4)
Medium (2–3)	42,741 (48.3)	7,821 (46.8)	17,252 (48.2)	12,755 (46.6)	4,913 (57.1)
High (4–5)	1,959 (2.2)	267 (1.6)	615 (1.7)	597 (2.2)	480 (5.6)
Body mass index, kg/m^2^ [mean (SD)]	26.67 (4.49)	26.73 (4.61)	26.48 (4.06)	26.15 (4.32)	28.93 (5.60)
Overall average acceleration (no-wear time bias adjusted), mg (median [IQR])	27.25 [22.59, 32.71]	28.87 [23.87, 34.99]	26.86 [22.60, 32.03]	28.46 [24.04, 33.68]	21.32 [16.75, 26.23]
Moderate-vigorous physical activity, minutes/week (median [IQR])	453.60 [310.69, 628.69]	497.92 [348.26, 686.58]	456.84 [320.76, 626.88]	478.76 [340.27, 646.65]	269.83 [161.28, 413.28]
Average acceleration per hour for 4 periods of 1 d, mg (median [IQR])					
Morning (06:00–07:59)	15.07 [7.32, 27.58]	39.48 [29.65, 52.62]	10.95 [6.06, 18.07]	15.29 [7.99, 24.52]	7.00 [3.90, 13.52]
Daytime (08:00–19:59)	42.05 [34.44, 50.93]	41.94 [34.38, 51.11]	44.04 [36.74, 52.94]	42.89 [35.91, 51.16]	30.17 [23.80, 36.96]
Evening (20:00–00:59)	16.15 [12.26, 21.36]	13.97 [10.81, 17.96]	13.77 [10.98, 17.16]	21.55 [17.36, 26.98]	17.47 [12.84, 23.88]
Night (01:00–05:59)	3.27 [2.77, 4.20]	3.92 [3.05, 5.82]	3.01 [2.64, 3.53]	3.15 [2.72, 3.77]	5.62 [4.26, 8.07]

SD, standard deviation; IQR, interquartile range; mg, milligravity

For PA intensity (Table 1), participants in the accelerometer-derived night-active phenotype were more likely to have lower total PA (bias-adjusted average acceleration for no-wear time) and higher PA intensity in the night period (01:00 to 05:59) compared with other types. Participants in the accelerometer-derived early-morning-active, daytime-active, and evening-active groups had higher PA intensity in the morning period (06:00 to 07:59), daytime period (08:00 to 19:59), and evening period (20:00 to 00:59), respectively.

### Accelerometer-derived rest–activity timing phenotypes and mental health

**Fig. 2.**
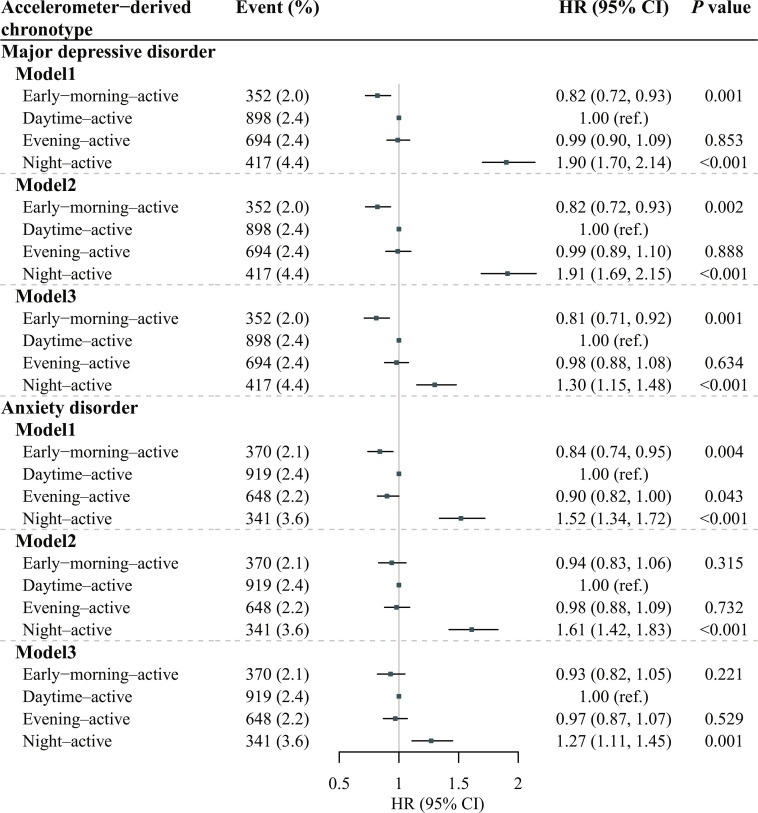
Associations of accelerometer-derived rest–activity timing phenotypes with mental disorders. Model 1 (basic) was an unadjusted model. Model 2 (sociodemographic) was adjusted for age at accelerometry test, gender, ethnicity, education level, and quintiles of TDI. Model 3 (sociodemographic and lifestyle) was adjusted for smoking status, alcohol drinking frequency, diet score, BMI, sleep score, and moderate-vigorous PA time, besides covariates in model 2. *P* values below the corrected threshold (0.05/2 = 0.025) were considered significant.

Participants were followed for a median of approximately 6.8 years (IQR: 6.2 to 7.3 years) from the accelerometry assessment (May 2013 to December 2015) to outcome occurrence or the end of follow-up, and we observed 2,361 MDD and 2,278 anxiety disorders. Compared with participants in the accelerometer-derived daytime-active group, those in the night-active group had higher risks of MDD (HR: 1.30, 95% CI: 1.15 to 1.48) and anxiety disorders (HR: 1.27, 95% CI: 1.11 to 1.45) (Fig. 2). A 19% reduction in the risk of MDD was observed for participants with the early-morning-active phenotype (95% CI: 8% to 29%). Comparing model 1 (basic) with models 2 (sociodemographic) and 3 (sociodemographic plus lifestyle) indicated that additional covariate adjustment generally produced only small changes in the estimated associations between accelerometer-derived rest–activity timing phenotypes and mental health.

There was a significant interaction effect between accelerometer-derived rest–activity timing phenotypes and age group for anxiety disorders in model 3 (*P* for multiplicative interaction = 0.008) (Fig. S3). Among participants aged <60 years, the protective effect of early-morning-active phenotype was more pronounced for anxiety disorders. Among participants aged ≥60 years, the adverse effect of night-active phenotype was more pronounced for anxiety disorders. Compared with daytime-active group, females in the night-active group had a 75% higher risk of MDD, while males in the early-morning-active and night-active groups had 6% and 35% lower risks of MDD in model 1 (*P* for multiplicative interaction = 0.046) (Fig. S4). There was no significant interaction effect between accelerometer-derived rest–activity timing phenotypes and PA level groups on mental health in any model (Fig. S5).

Sensitivity analyses verified the robustness of the main findings. In the 1- and 2-year landmark analyses, the associations of accelerometer-derived rest–activity timing phenotypes with mental health were attenuated (Figs. S6 and S7). Among complete cases (participants with no missing values), the magnitudes of associations were larger than the main analysis for anxiety disorders. The associations of accelerometer-derived rest–activity timing phenotypes with MDD were lower than the main analysis for outcomes (Fig. S8). The *E* values for the observed associations ranged from 1.51 (for the association of the early-morning-active group with anxiety disorders in model 1) to 2.50 (for the association of the night-active group with MDD in model 2) (Fig. S9). Results were materially unchanged in additional sensitivity analyses excluding baseline major psychiatric comorbidities (Fig. S10), restricting to participants without current shift work/night shift work (Fig. S11), and restricting to those without baseline sleep disorders (Fig. S12). Effect estimates were also similar in extended models additionally adjusting for prevalent hypertension, diabetes, cancer, and sleep disorders (Fig. S13), as well as when restricting analyses to the MRI subcohort (Fig. S14), supporting the robustness of the observed associations.

### Measured PA intensity of each hour and mental health

**Fig. 3.**
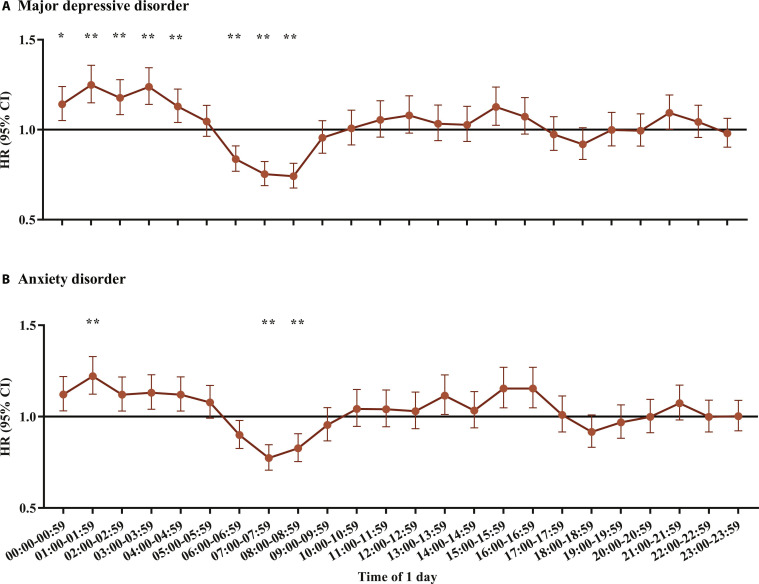
Associations of PA intensity over the 24-h cycle with mental disorders. (A) Hazard ratios for incident major depressive disorder by hourly physical activity intensity over the 24-h cycle. (B) Hazard ratios for incident anxiety disorders by hourly physical activity intensity over the 24-h cycle. HRs and 95% CIs were from Cox models comparing high and low PA levels (using the median as the cutoff point) of each 1-h period of 24-h cycle adjusted for age at accelerometry test, gender, ethnicity, education level, quintiles of TDI, smoking status, alcohol drinking frequency, diet score, BMI, sleep score, and moderate-vigorous PA time. *P values were below the multi-comparisons threshold of 0.05/24 = 0.00208. **P values were below the multi-comparisons threshold of 0.05/(24*10) = 0.000208. The cutoff points of each hour are listed in Table S2.

Figure 3 shows the associations of PA intensity of each hour over the 24-h cycle with mental disorders. The hazards and benefits of high PA levels were most pronounced in the period of 0:00 to 8:59, that is, the night and early morning periods. In the period of 00:00 to 04:59, high PA levels of each hour defined by PA intensity above the median were associated with higher risks of MDD and anxiety disorders, compared with low PA levels defined by PA intensity at or below the median. In the period of 06:00 to 08:59, high PA levels were associated with lower risks of MDD and anxiety disorders compared with low PA levels. In sensitivity analysis, the results were mostly consistent (Figs. S15 and S16).

### Accelerometer-derived rest–activity timing phenotypes and brain MRI indices

The results of the multiple regression analysis assessing accelerometer-derived rest–activity timing phenotypes in relation to brain MRI indices are shown in Table 2. Compared with the daytime-active group, the night-active group showed a −0.077 SD difference in total brain (GM and WM) volume (SE: 0.024), along with lower frontoparietal GM volumes (including the frontal pole and precuneus cortex) and reduced subcortical volumes (thalamus, putamen, pallidum, and hippocampus). In contrast, measures reflecting WM tract integrity did not differ significantly between groups. These MRI findings should be interpreted as exploratory correlates within the imaging subset.

**Table 2.** Association between accelerometer-derived rest–activity timing phenotypes and brain structure and volume ^a^ (*N* = 17,571). Bold type indicates statistical significance. Multiple linear models adjusted for age at accelerometry test, gender, ethnicity, education level, quintiles of TDI, smoking status, alcohol drinking frequency, diet score, BMI, sleep score, moderate-vigorous physical activity time, scanner table position, and scanner brain position.

Brain MRI indices	Early-morning-active (*n* = 7,149)		Daytime-active (*n* = 3,346)	Evening-active (*n* = 5,600)		Night-active (*n* = 1,476)	
	β (SE)	*P* value ^b^		β (SE)	*P* value ^b^	β (SE)	*P* value ^b^
Brain atrophy							
GM ^c^	−0.045 (0.016)	0.076	Reference	0.004 (−0.009)	0.777	−0.058 (0.022)	**0.022**
WM ^c^	−0.027 (0.020)	0.454	Reference	0.191 (−0.003)	0.872	−0.074 (0.028)	**0.022**
GM + WM ^c^	−0.044 (0.017)	0.114	Reference	0.012 (−0.007)	0.798	−0.077 (0.024)	**0.006**
Frontoparietal GM							
Frontal pole ^c^	0.006 (0.018)	0.843	Reference	0.754 (0.029)	0.224	−0.055 (0.024)	**0.049**
Superior frontal gyrus ^c^	0.007 (0.019)	0.841	Reference	0.708 (0.037)	0.399	0.000 (0.025)	0.992
Middle temporal gyrus (temporooccipital) ^c^	0.031 (0.017)	0.352	Reference	0.074 (0.011)	0.829	−0.05 (0.024)	0.068
Angular gyrus ^c^	0.024 (0.017)	0.470	Reference	0.173 (0.013)	0.796	−0.031 (0.024)	0.238
Cingulate gyrus ^c^	−0.019 (0.019)	0.602	Reference	0.317 (0.033)	0.176	−0.031 (0.025)	0.255
Precuneus cortex ^c^	0.008 (0.019)	0.863	Reference	0.681 (0.017)	0.801	−0.094 (0.026)	**<0.001**
WMH ^a^	0.028 (0.020)	0.507	Reference	0.16 (0.013)	0.876	0.002 (0.027)	0.986
White matter tract integrity							
gFA	−0.014 (0.021)	0.732	Reference	0.501 (−0.011)	0.757	−0.039 (0.03)	0.254
gMD	0.015 (0.021)	0.741	Reference	0.468 (0.008)	0.785	0.04 (0.028)	0.258
Subcortical volumes							
Thalamus ^c^	−0.025 (0.018)	0.581	Reference	0.153 (0.032)	0.222	−0.091 (0.024)	**<0.001**
Caudate ^c^	−0.012 (0.02)	0.732	Reference	0.539 (0.016)	0.822	−0.054 (0.027)	0.081
Putamen ^c^	0.002 (0.017)	0.920	Reference	0.92 (0.034)	0.228	−0.063 (0.024)	**0.021**
Pallidum ^c^	−0.043 (0.019)	0.152	Reference	0.024 (0.01)	0.798	−0.079 (0.026)	**0.010**
Hippocampus ^c^	−0.022 (0.018)	0.479	Reference	0.227 (0.006)	0.753	−0.073 (0.025)	**0.011**
Amygdala ^c^	0.002 (0.017)	0.952	Reference	0.902 (0.024)	0.348	−0.018 (0.023)	0.492
Accumbens ^c^	−0.016 (0.017)	0.620	Reference	0.359 (0.006)	0.792	−0.033 (0.024)	0.240

gFA/gMD, standardized first principal component scores of white matter fractional anisotropy and mean diffusivity; WMH, white matter hyperintensity; GM/WM/GM + WM, volumes of gray matter, white matter, and their sum; SE, standard error; MRI, magnetic resonance imaging; β, beta coefficient

^a^ Log-transformed (offset = 1).

^b^ *P* values were adjusted for the false discovery rate.

^c^ Brain volume indicators were converted to *Z* scores.

## Discussion

Using a large-scale cohort from the UK Biobank, this study identified 4 measured rest–activity timing phenotypes from wrist-worn accelerometry data using *k*-means clustering analysis. The accelerometer-derived night-active group had a 30% higher risk of MDD and a 27% higher risk of anxiety disorders compared with daytime-actives group. Participants in the early-morning-active group had a 19% lower risk of MDD. Further, we found that high PA intensity defined by the median cutoff point in the night period (00:00 to 04:59) was associated with higher risks of MDD and anxiety disorders, whereas high PA intensity in the morning period (06:00 to 08:59) was associated with lower risks of MDD and anxiety disorders. The observed MRI correlates may be consistent with neurobiological pathways linked to mental health, but they do not establish mediation or causality. Collectively, these findings indicate that activity timing phenotypes derived from wearables are prospectively associated with common mental disorders and exhibit exploratory neuroimaging correlates, supporting their potential utility as behavioral markers in population health research.

In our study, we identified 4 accelerometer-derived rest–activity timing phenotypes using 24-h activity profiles. These phenotypes are descriptive behavioral patterns in free-living settings: The early-morning-active phenotype showed relatively higher activity between 06:00 and 07:59, whereas the night-active phenotype showed relatively higher activity between 01:00 and 05:59 compared with other groups. Importantly, these labels describe relative differences in activity timing rather than questionnaire-defined chronotype categories, and they should not be interpreted as direct measures of endogenous circadian phase. Elevated nocturnal activity may also reflect externally constrained schedules (e.g., shift work), habitual behaviors, or sleep problems, and we therefore examined results after excluding participants reporting shift/night-shift work. Importantly, wrist-worn accelerometry captures rest–activity timing phenotypes in free-living settings. These phenotypes provide an objective and ecologically valid indicator of behavioral chronotype, but they may reflect the combined influences of endogenous circadian regulation as well as work/school schedules and habitual behaviors. Therefore, the accelerometer-derived rest–activity timing phenotypes identified here are best interpreted as a behavioral phenotypes related to, but not equivalent to, endogenous circadian phase or amplitude [28,39].

We observed that incident MDD and anxiety disorders were associated with accelerometer-derived rest–activity timing phenotypes independent of traditional risk factors. Our findings are consistent with the existing literature on chronotype and mental health [12,40,41]. Previous studies on the mental health of chronotypes mainly derived chronotypes using the self-report method like questionnaires [12,41]. According to these studies, self-reported evening chronotype was independently associated with severe psychiatric symptoms compared to morning chronotype [12,40,41]. Studies also reported that the morning chronotype may be protective against psychiatric symptoms [12,40,41]. Furthermore, limited studies have evaluated the mental health of measured behavioral chronotypes [12].

In addition, accelerometer-derived rest–activity timing phenotypes were cross-sectionally associated with several MRI-derived brain structural measures; these findings are hypothesis-generating and should not be interpreted causally. Prior research has linked specific MRI measures to chronotype, which may help clarify neurobiological biomarkers relevant to mental health [42–44]. In this study, we examined whether MRI indices relevant to mental health differed across accelerometer-derived behavioral chronotype groups. Individuals classified as night-active exhibited greater reductions in total and frontoparietal GM. Such GM alterations may be particularly relevant to mental health status [44]. These structural differences may represent one pathway linking disrupted activity patterns to mental health risk. In line with this mechanistic perspective, a recent study found that the brain age gap partially mediated the association between higher vigorous PA and incident depressive disorder, suggesting that accelerated brain aging could be a common neurobiological pathway through which different aspects of daily activity—including its timing and intensity—influence depression risk [45]. Consistent with previous studies, reduced hippocampal volume and decreases in other subcortical structures were also observed among night-active participants [43].

Some underlying mechanisms could contribute to the interpretation of these identified associations. Firstly, late activity timing may entail greater exposure to artificial light at night (ALAN). Recent evidence, including Deprato et al. [46], suggests that exposure to ALAN is independently associated with adverse mental health outcomes, particularly depression; this pathway may represent both a mechanistic contributor and a source of residual confounding that warrants direct measurement in future studies. Secondly, self-reported chronotype (morningness/eveningness) correlates with differences in sleep–wake schedules and may also relate to downstream behaviors and physiology, including meal timing and eating behaviors, digestion, body temperature, heart rate, and mood [47,48]. All of these behaviors and physiological biomarkers are crucial to immune function, homeostasis, and metabolism [48]. Thirdly, late chronotype individuals might have adverse lifestyle behaviors, including unhealthy eating habits and low sleep quality measured by the Pittsburgh Sleep Quality Index [49,50]. Fourthly, chronotypes also potentially influence metabolic profiles [51]. The analysis based on 4 cohort studies across different cultural, environmental, and genetic backgrounds suggested that evening types were associated with higher emotional eating scores and were at increased risk of obesity and metabolic disorders [40,52,53]. Evening chronotypes were associated with elevated circulating proteins related to insulin resistance and cardiometabolic risks, and higher levels of inflammation, which are prodromal or intermediately involved in the etiopathogenesis of multiple diseases [52,53]. In addition, growing evidence supports that diurnal oscillations in the gut microbiota composition and function exert profound influences on circadian biology and human well-being [40]. However, the precise mechanisms involved in disease-specific pathways remain elusive and need further research.

Our results highlight rest–activity timing phenotypes as potentially modifiable behavioral correlates of mental health; whether changing activity timing reduces incident disorder risk requires randomized or quasi-experimental evidence. With the increasing adoption of modern lifestyles, artificial light exposure, and industrialization, disruption of circadian timing and daily rest–activity rhythms is progressively emerging as a prevalent phenomenon. Chronic shift work is a major contributor to circadian misalignment and disrupted rest–activity timing phenotypes, and is linked to an elevated risk of poor mental health [54]. Therefore, it is necessary to pay attention to circadian rhythm issues. Chronotherapeutic approaches aim to restore physiological circadian timing and strengthen daily rhythmicity through temporal regulation of feeding and fasting, light therapy, regular activities, and melatonin prescription [40,55]. Studies have suggested that resynchronization of circadian rhythms could commonly improve psychiatric symptoms [40,55]. As suggested by our findings, wearable devices such as accelerometers may provide objective monitoring of rest–activity timing phenotypes that could support risk evaluation and potentially facilitate chronotherapy implementation; however, these measures should be interpreted as behavioral proxies rather than direct markers of endogenous circadian function.

With a large sample from the UK Biobank cohort, we prospectively assessed the association of accelerometer-derived rest–activity timing phenotypes and mental health. Using wrist-worn accelerometry, we derived rest–activity timing phenotypes using *k*-means clustering analysis. Using multidimensional exposure data from the well-characterized cohort, traditional risk factors and confounders were fully adjusted for. The MRI subset (*n* = 17,571) enabled exploratory analyses of brain structural correlates of these activity timing phenotypes. There were some limitations in this study. A key limitation is the reliance on ICD-10 codes for case identification, which introduces selection bias through the exclusion of undiagnosed or untreated individuals. Consequently, our findings lack generalizability beyond the severe, treatment-seeking end of the clinical spectrum. Critically, this bias would attenuate the observed effect because the exclusion of any undiagnosed cases sharing this association dilutes the signal, biasing the HR toward the null. Thus, our reported association is likely a conservative estimate, and its detection despite this attenuation reinforces the finding. The neuroimaging analyses were conducted in a subset of participants with available MRI data, which may introduce selection bias. However, the consistency of our findings across different analytical samples provides some reassurance regarding the robustness of the observed associations. Nevertheless, replication in independent cohorts with broader imaging coverage is warranted. Our accelerometer-derived rest–activity timing phenotypes primarily reflect phase-related timing patterns, and we did not quantify rhythm amplitude or day-to-day stability; given evidence that rhythm amplitude/robustness may be highly relevant to in mental disorders [56], future studies should integrate amplitude- and stability-related rhythm metrics alongside timing phenotypes. Individuals’ activity timing patterns may shift across the lifespan. A single accelerometry assessment may fail to fully capture long-term habitual patterns of activity timing across the life course. However, because participants were middle-aged to older adults, their habitual activity timing patterns may be relatively stable compared with earlier life stages [20]. Many covariates in this study were captured at the baseline visit, which in some cases was several years before accelerometry. Thus, the potential for misclassification in covariates was unavoidable. Furthermore, UK Biobank participants are recognized as being healthier than the general population, reflecting a “healthy volunteer” selection bias, which may limit external generalizability [57]. This selection may affect absolute risk estimates and the representativeness of the sample; however, exposure–outcome associations are often less sensitive to nonrepresentativeness [57]. Although traditional risk factors and confounders were all adjusted for in the regression models, residual confounding of unmeasured factors still could not be fully ruled out. Nonetheless, our results were robust across multiple sensitivity analyses (complete-case analyses, 1- and 2-year landmark analyses, and *E*-value calculations), supporting the internal validity of the observed associations.

## Conclusion

Among over 90,000 UK Biobank participants with accelerometer data enrolled in this prospective cohort study, we identified 4 distinct activity timing phenotypes. Compared with the daytime-active phenotype, the night-active phenotype was associated with higher hazards of incident MDD and anxiety disorders, whereas the early-morning-active phenotype was associated with a lower hazard of MDD. Neuroimaging findings in a subset of participants provided exploratory correlates that may help generate mechanistic hypotheses. Our findings suggest that objectively measured activity timing phenotypes may serve as a behavioral marker for mental health risk stratification. However, whether modification of activity timing through behavioral interventions could reduce mental disorder risk requires investigation in randomized controlled trials.

## Ethical Approval

The UK Biobank received ethical approval from the National Information Governance Board for Health and Social Care and the National Health Service North West Multi-Center Research Ethics Committee. All participants gave informed consent through electronic signature before enrollment in the study. Analyses were conducted under application number 45676. This research conforms to the Declaration of Helsinki.

## Data Availability

The UK Biobank resource is open to all researchers (https://www.ukbiobank.ac.uk). Statistical code is available on request via email from the corresponding authors.
