# Supplementary material for: Rest–Activity Timing Phenotypes and Mental Health: A Longitudinal Analysis of 24-Hour Accelerometry in Population-Based Cohorts
Source: Health Data Sci. 2026 Mar 3;6:0306. doi: 10.34133/hds.0306 (PMC12953921; doi:10.34133/hds.0306)
Supplement: Supplementary 1 — Figs. S1 to S16 Tables S1 to S5 [file hds.0306.f1.docx]

**Supplemental materials**

**Title: Rest-activity timing phenotypes and mental health: a longitudinal analysis of 24-hour accelerometry in population-based cohorts**

Content

[**Figure S1** Participant selection flowchart. 3](#_Toc218015718)

[**Figure S2** Within groups sums of squares of different cluster numbers. 4](#_Toc218015719)

[**Figure S3** Association of accelerometer-derived chronotype with mental disorders among participants aged < 60 years and ≥ 60 years. 5](#_Toc218015720)

[**Figure S4** Association of accelerometer-derived rest-activity timing phenotypes with mental disorders among males and females. 6](#_Toc218015721)

[**Figure S5** Association of accelerometer-derived rest-activity timing phenotypes with mental disorders among participants with low or high PA level. 7](#_Toc218015722)

[**Figure S6** Association of accelerometer-derived rest-activity timing phenotypes with mental disorders using 1-year landmark analysis (N=92,986). 8](#_Toc218015723)

[**Figure S7** Association of accelerometer-derived rest-activity timing phenotypes with mental disorders using 2-year landmark analysis (N=90,776). 9](#_Toc218015724)

[**Figure S8** Association of accelerometer-derived rest-activity timing phenotypes with mental disorders among participants with no missing value in covariates (N=71,853). 10](#_Toc218015725)

[**Figure S9** E-values for the associations of accelerometer-derived rest-activity timing phenotypes with mental disorders. 11](#_Toc218015726)

[**Figure S10** Association of accelerometer-derived rest-activity timing phenotypes with mental disorders: excluding participants with baseline bipolar disorder, psychotic disorders, ADHD, and substance use disorders (N=94,179). 12](#_Toc218015727)

[**Figure S11** Association of accelerometer-derived rest-activity timing phenotypes with mental disorders among participants who did not report current shift work or night shift work at baseline (N=86,699). 13](#_Toc218015728)

[**Figure S12** Association of accelerometer-derived rest-activity timing phenotypes with mental disorders among participants without sleep disorders at baseline (N=93,340). 14](#_Toc218015729)

[**Figure S13** Extended models additionally adjusting for prevalent chronic conditions (hypertension, diabetes, and cancer) and sleep disorders (N=94,344). 15](#_Toc218015730)

[**Figure S14** Association of accelerometer-derived rest-activity timing phenotypes with mental disorders restricted to the MRI subcohort (N=17,571). 16](#_Toc218015731)

[**Figure S15** Associations of PA intensity over the 24-hour cycle with mental disorders using 2-year landmark analysis (N=90,776). 17](#_Toc218015732)

[**Figure S16** Associations of PA intensity over the 24-hour cycle with mental disorders among participants with no missing value in covariates (N=71,853). 18](#_Toc218015733)

[**Table S1** The description of physical activity intensity in each hour period over the 24-hour cycle. 19](#_Toc218015734)

[**Table S2** Covariate definitions in multivariate regression models. 20](#_Toc218015735)

[**Table S3** Tract loadings on the first principal component of white matter fractional anisotropy (gFA) and mean diffusivity (gMD) 21](#_Toc218015736)

[**Table S4** Baseline characteristics of participants with vs without MRI data 22](#_Toc218015737)

[**Table S5** Definitions of comorbidities and shift-work variables 24](#_Toc218015738)

UK Biobank participants

with accelerometer data

N = 103,683

N = 96,684

Excluded 6,999 participants with < 3 days of wear, absent data in each one-hour period of the 24-hour cycle, poor calibration, or implausible average acceleration (> 500 milli-g)

N = 94,367

Excluded 2,340 participants diagnosed with outcomes of interest at or before baseline

Excluded 23 participants without age and gender information

N = 94,344 included in measured chronotypes analysis

N = 17,571 included in brain MRI analysis

Excluded 76,773 participants without brain MRI information

**Figure S1** Participant selection flowchart.


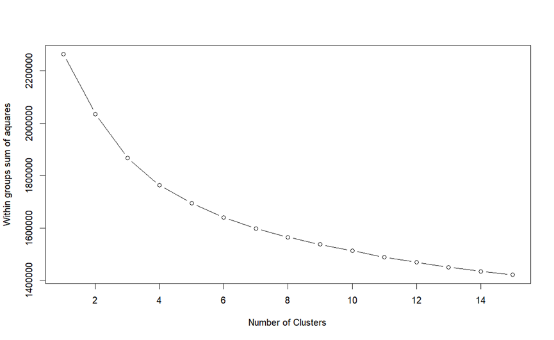


**Figure S2** Within groups sums of squares of different cluster numbers.

The within groups sum of squares decreased slowly with 4 and more clusters. Thus, 4 was the best number of clusters.


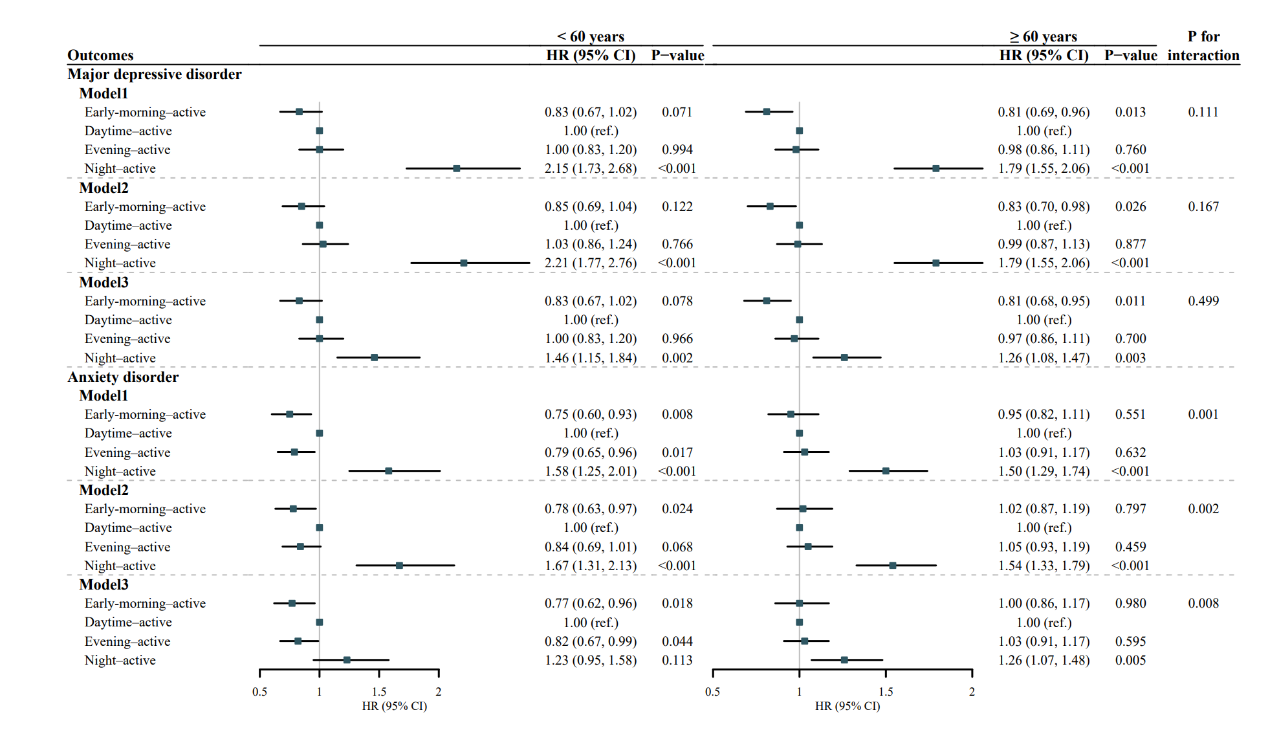


**Figure S3** Association of accelerometer-derived chronotype with mental disorders among participants aged < 60 years and ≥ 60 years.

Model 1 (basic) was unadjusted model. Model 2 (sociodemographic) was adjusted for age at accelerometry test, gender, ethnicity, education level, quintiles of TDI. Model 3 (sociodemographic and lifestyle) was adjusted for smoking status, alcohol drinking frequency, diet score, BMI, sleep score, moderate-vigorous physical activity time, besides covariates in model 2.


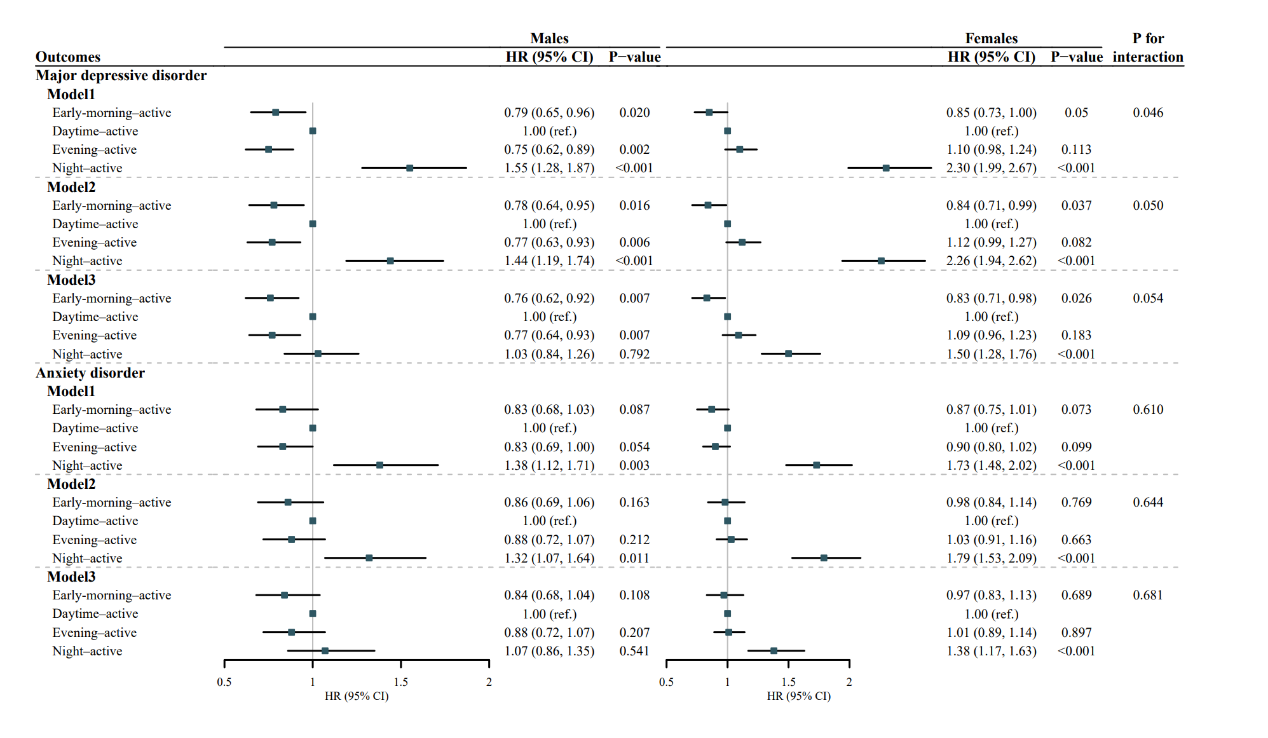


**Figure S4** Association of accelerometer-derived rest-activity timing phenotypes with mental disorders among males and females.

Model 1 (basic) was unadjusted model. Model 2 (sociodemographic) was adjusted for age at accelerometry test, gender, ethnicity, education level, quintiles of TDI. Model 3 (sociodemographic and lifestyle) was adjusted for smoking status, alcohol drinking frequency, diet score, BMI, sleep score, moderate-vigorous physical activity time, besides covariates in model 2.


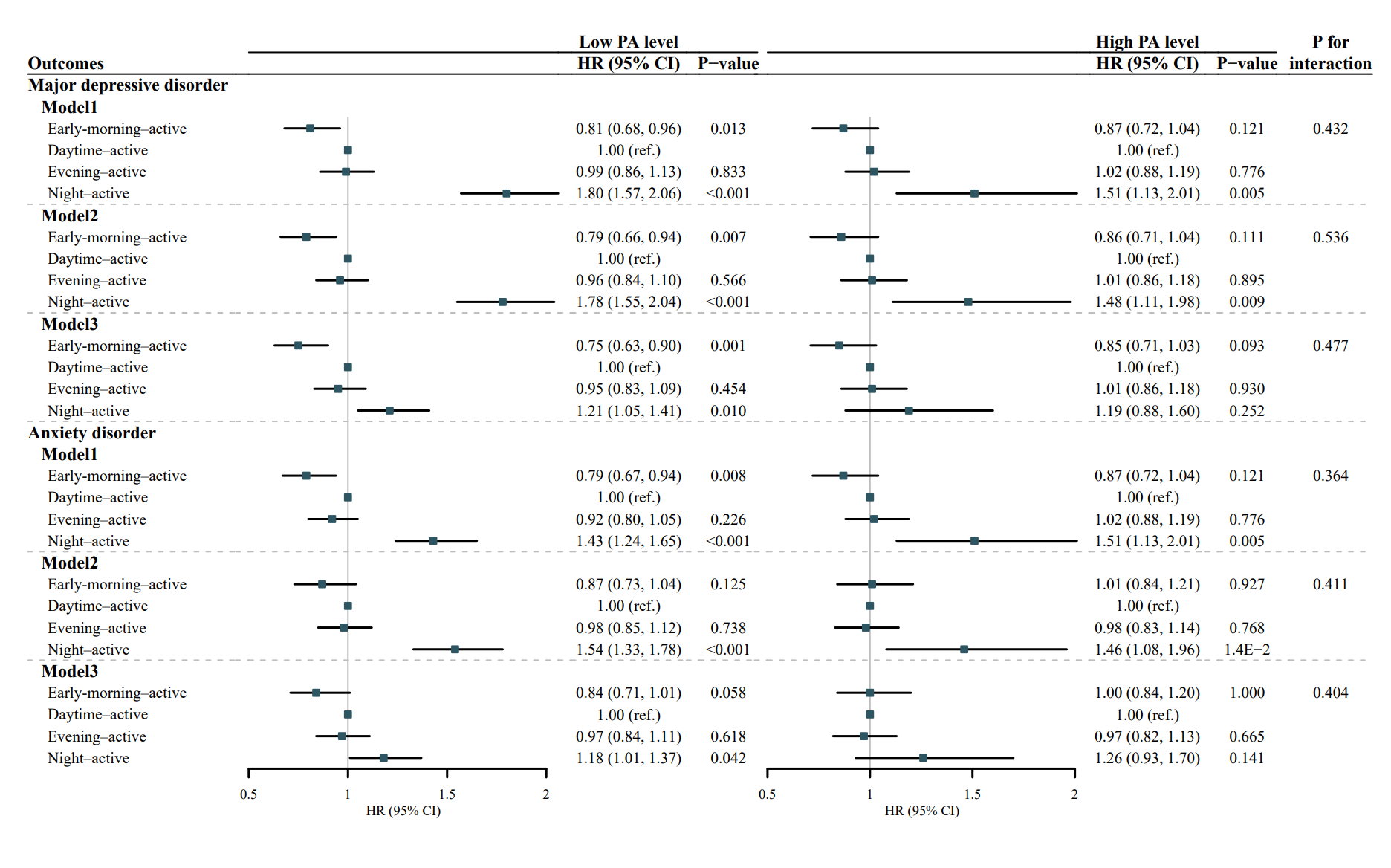


**Figure S5** Association of accelerometer-derived rest-activity timing phenotypes with mental disorders among participants with low or high PA level.

Model 1 (basic) was unadjusted model. Model 2 (sociodemographic) was adjusted for age at accelerometry test, gender, ethnicity, education level, quintiles of TDI. Model 3 (sociodemographic and lifestyle) was adjusted for smoking status, alcohol drinking frequency, diet score, BMI, sleep score, moderate-vigorous physical activity time, besides covariates in model 2. Low and high PA levels were defined using median cut-off point on Moderate-vigorous physical activity (470 minutes/week)


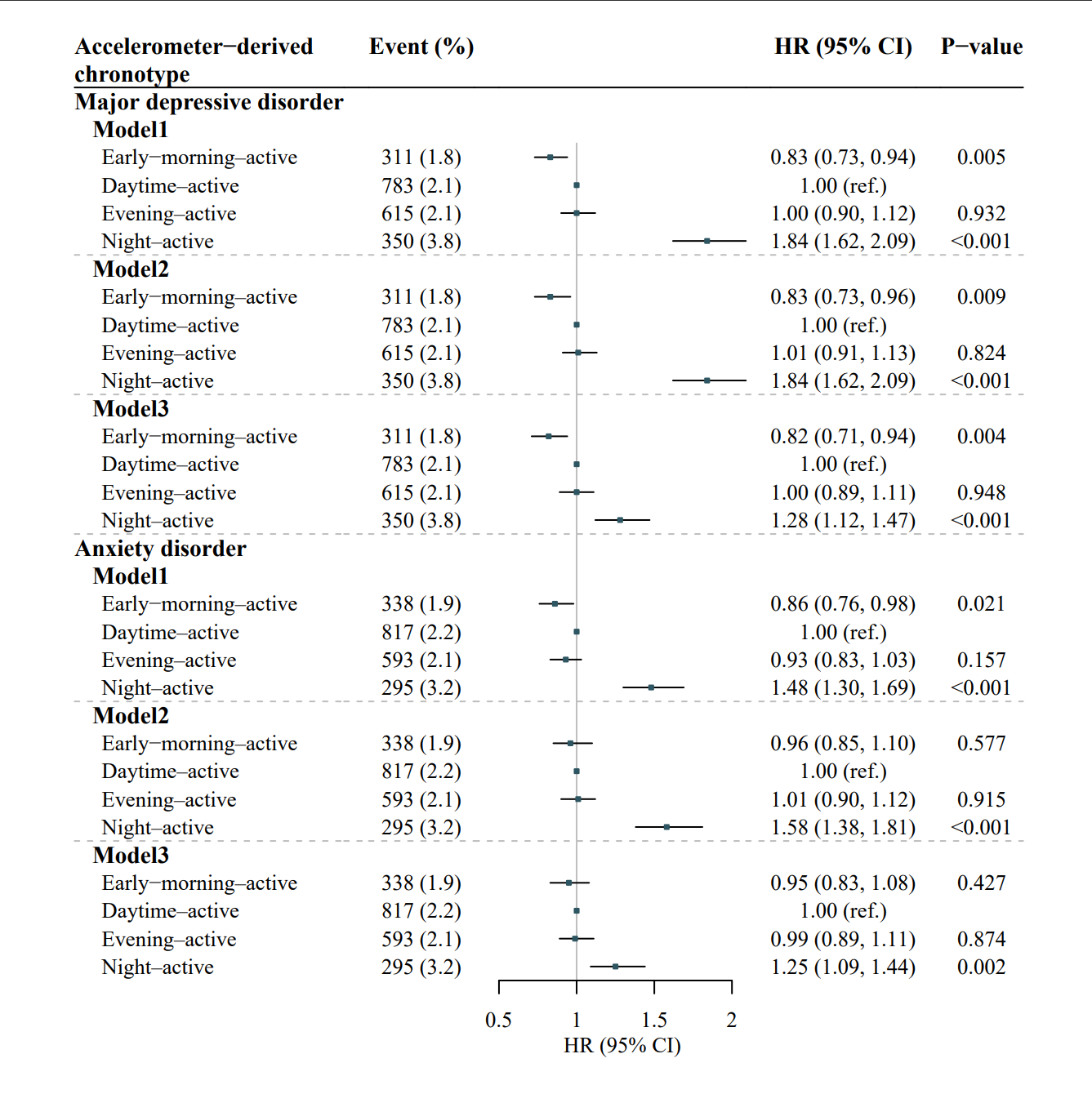


**Figure S6** Association of accelerometer-derived rest-activity timing phenotypes with mental disorders using 1-year landmark analysis (N=92,986).

Model 1 (basic) was unadjusted model. Model 2 (sociodemographic) was adjusted for age at accelerometry test, gender, ethnicity, education level, quintiles of TDI. Model 3 (sociodemographic and lifestyle) was adjusted for smoking status, alcohol drinking frequency, diet score, BMI, sleep score, moderate-vigorous physical activity time, besides covariates in model 2.


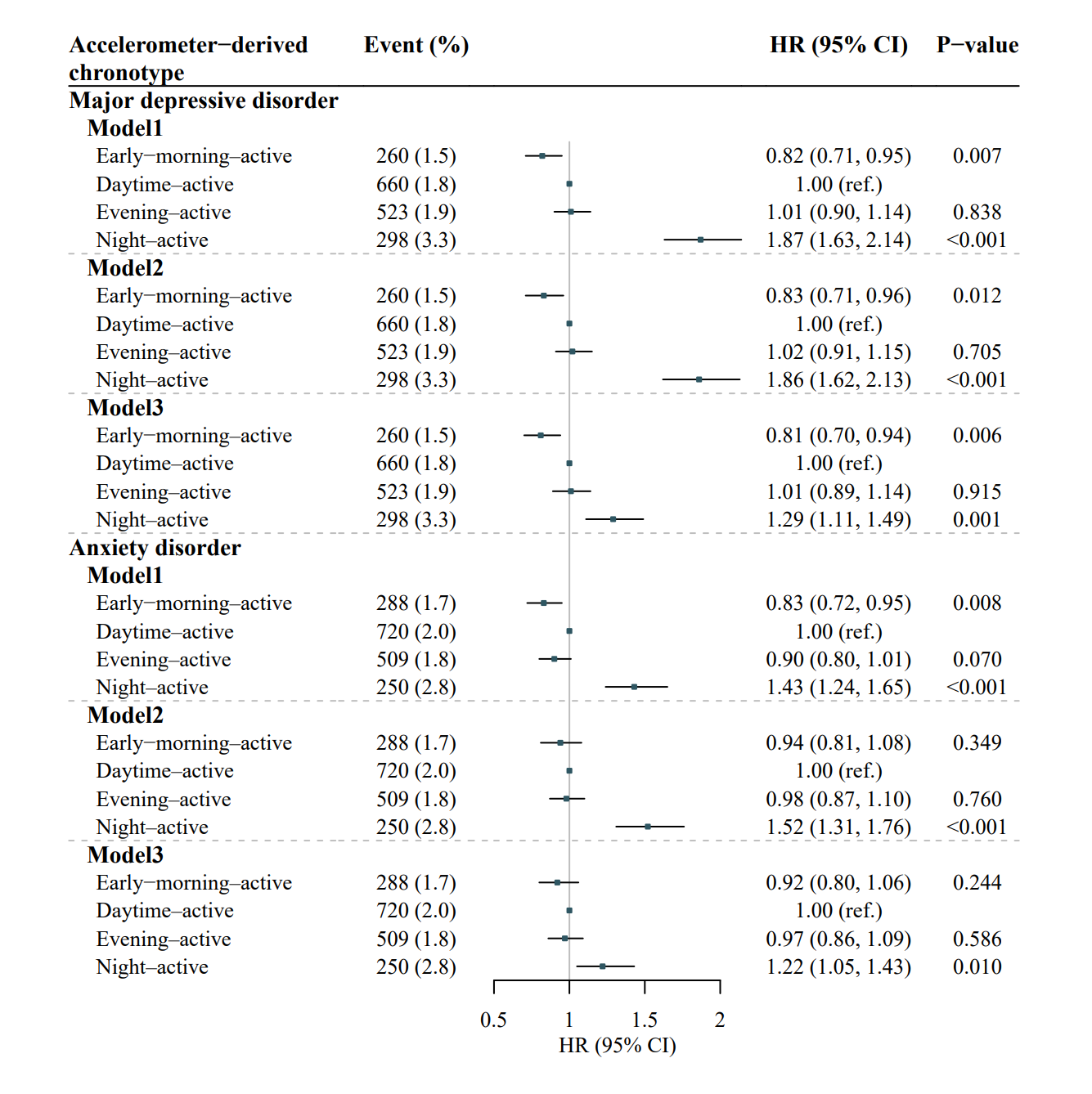


**Figure S7** Association of accelerometer-derived rest-activity timing phenotypes with mental disorders using 2-year landmark analysis (N=90,776).

Model 1 (basic) was unadjusted model. Model 2 (sociodemographic) was adjusted for age at accelerometry test, gender, ethnicity, education level, quintiles of TDI. Model 3 (sociodemographic and lifestyle) was adjusted for smoking status, alcohol drinking frequency, diet score, BMI, sleep score, moderate-vigorous physical activity time, besides covariates in model 2.

***
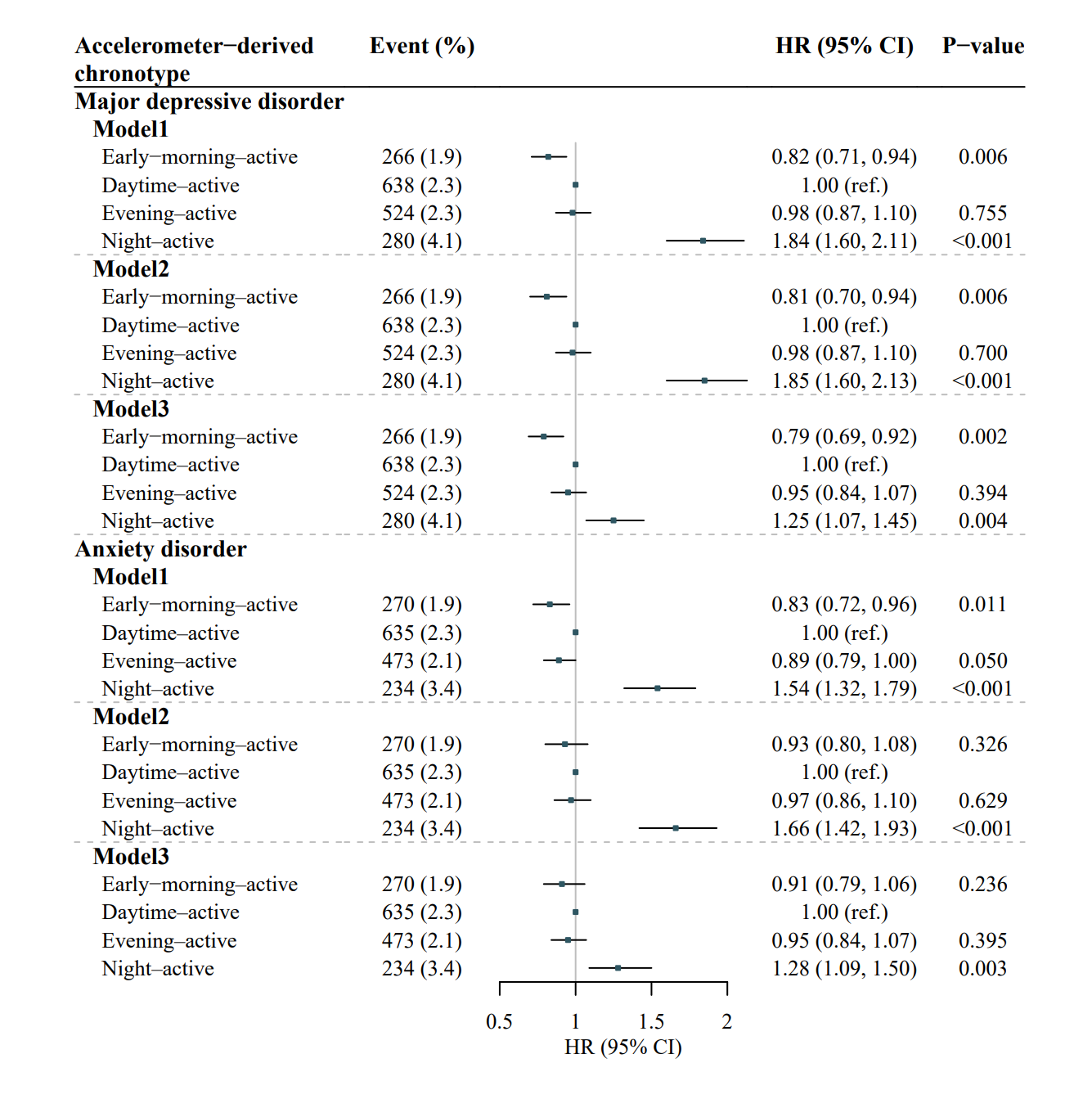
***

**Figure S8** Association of accelerometer-derived rest-activity timing phenotypes with mental disorders among participants with no missing value in covariates (N=71,853).

Model 1 (basic) was unadjusted model. Model 2 (sociodemographic) was adjusted for age at accelerometry test, gender, ethnicity, education level, quintiles of TDI. Model 3 (sociodemographic and lifestyle) was adjusted for smoking status, alcohol drinking frequency, diet score, BMI, sleep score, moderate-vigorous physical activity time, besides covariates in model 2.


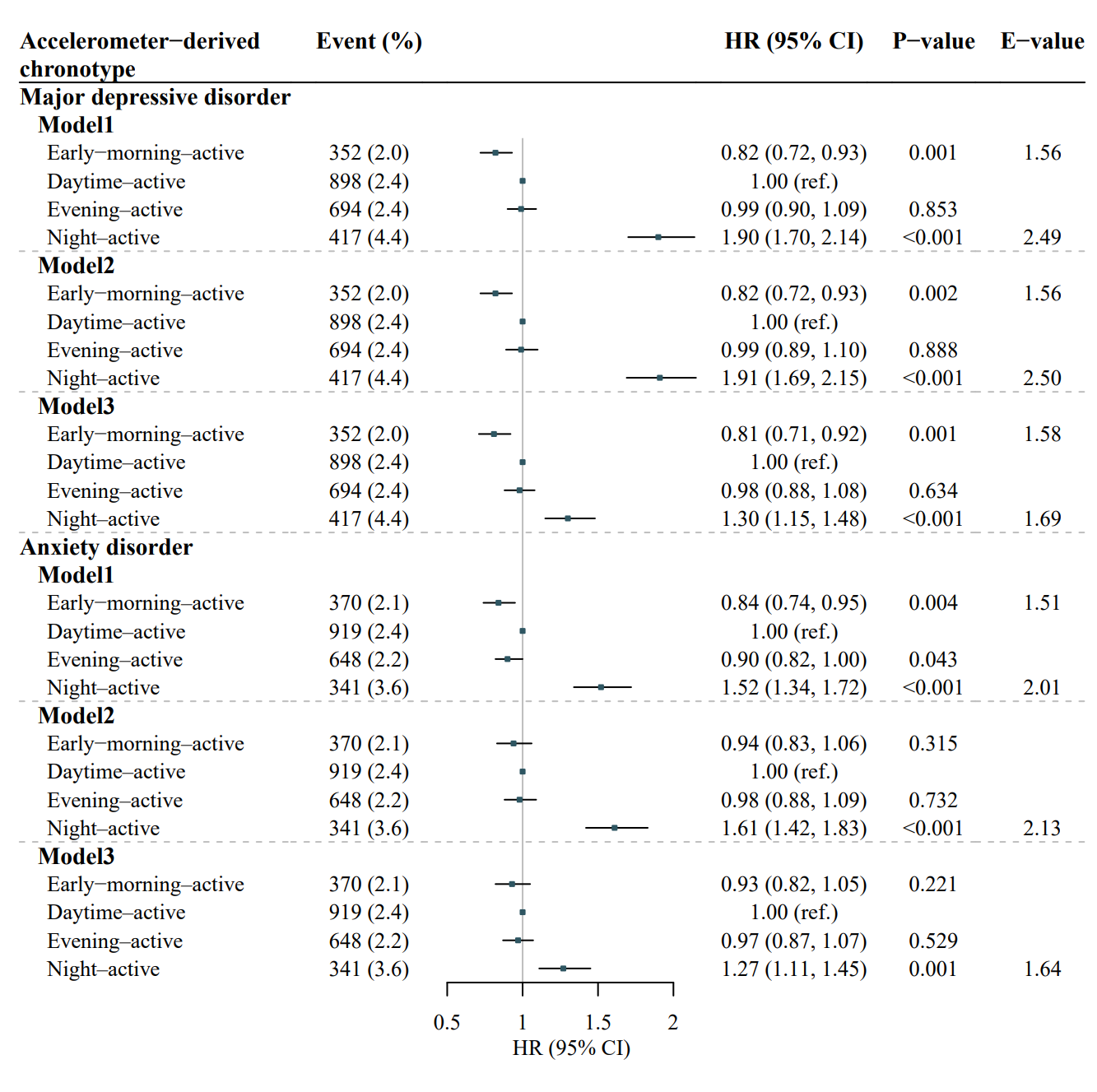


**Figure S9** E-values for the associations of accelerometer-derived rest-activity timing phenotypes with mental disorders.

Model 1 (basic) was unadjusted model. Model 2 (sociodemographic) was adjusted for age at accelerometry test, gender, ethnicity, education level, quintiles of TDI. Model 3 (sociodemographic and lifestyle) was adjusted for smoking status, alcohol drinking frequency, diet score, BMI, sleep score, moderate-vigorous physical activity time, besides covariates in model 2.

**
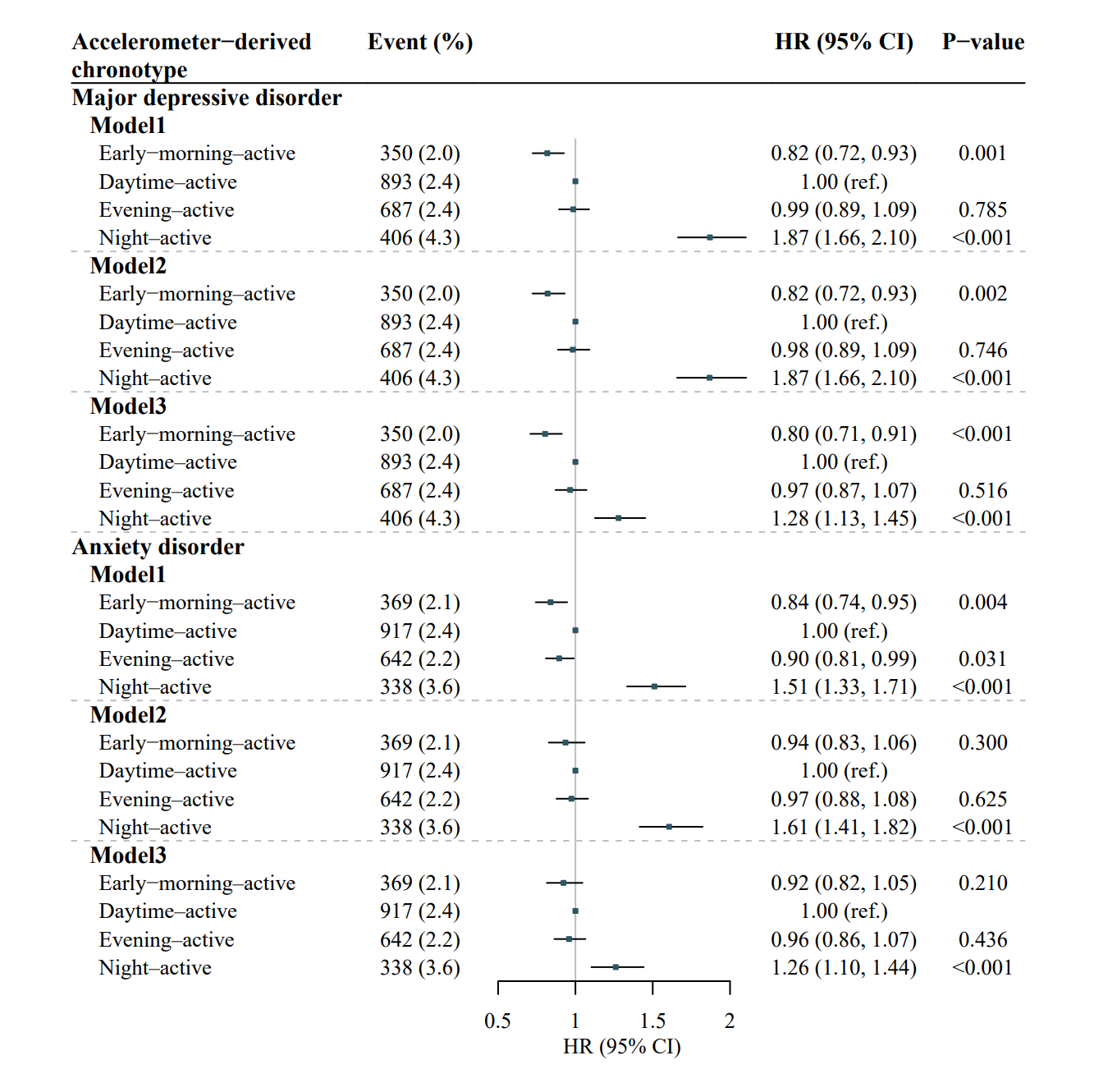
Figure S10** Association of accelerometer-derived rest-activity timing phenotypes with mental disorders: excluding participants with baseline bipolar disorder, psychotic disorders, ADHD, and substance use disorders (N=94,179).

Model 1 (basic) was unadjusted model. Model 2 (sociodemographic) was adjusted for age at accelerometry test, gender, ethnicity, education level, quintiles of TDI. Model 3 (sociodemographic and lifestyle) was adjusted for smoking status, alcohol drinking frequency, diet score, BMI, sleep score, moderate-vigorous physical activity time, besides covariates in model 2. ADHD: Attention Deficit Hyperactivity Disorder.

**
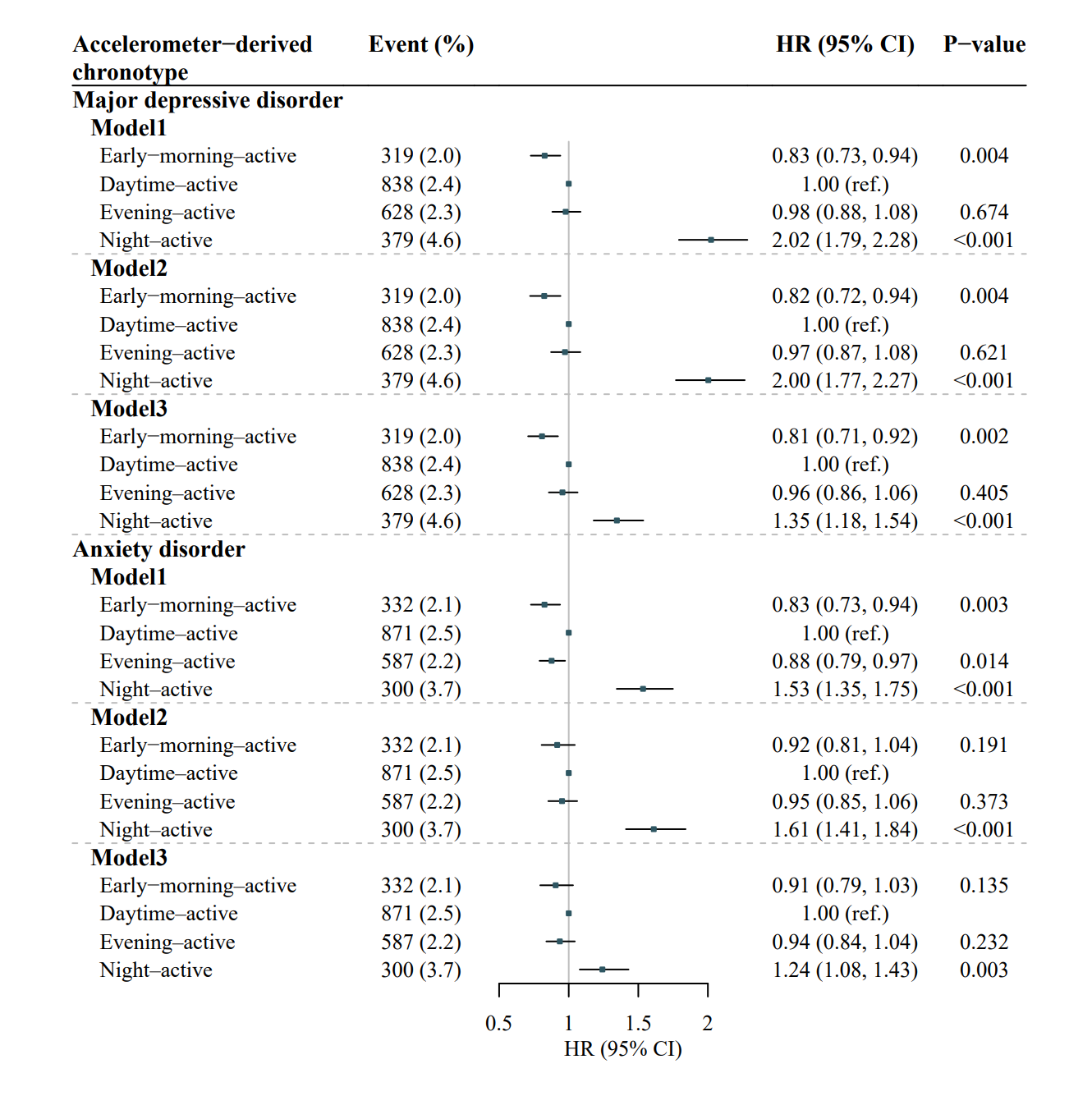
Figure S11** Association of accelerometer-derived rest-activity timing phenotypes with mental disorders among participants who did not report current shift work or night shift work at baseline (N=86,699).

Model 1 (basic) was unadjusted model. Model 2 (sociodemographic) was adjusted for age at accelerometry test, gender, ethnicity, education level, quintiles of TDI. Model 3 (sociodemographic and lifestyle) was adjusted for smoking status, alcohol drinking frequency, diet score, BMI, sleep score, moderate-vigorous physical activity time, besides covariates in model 2.


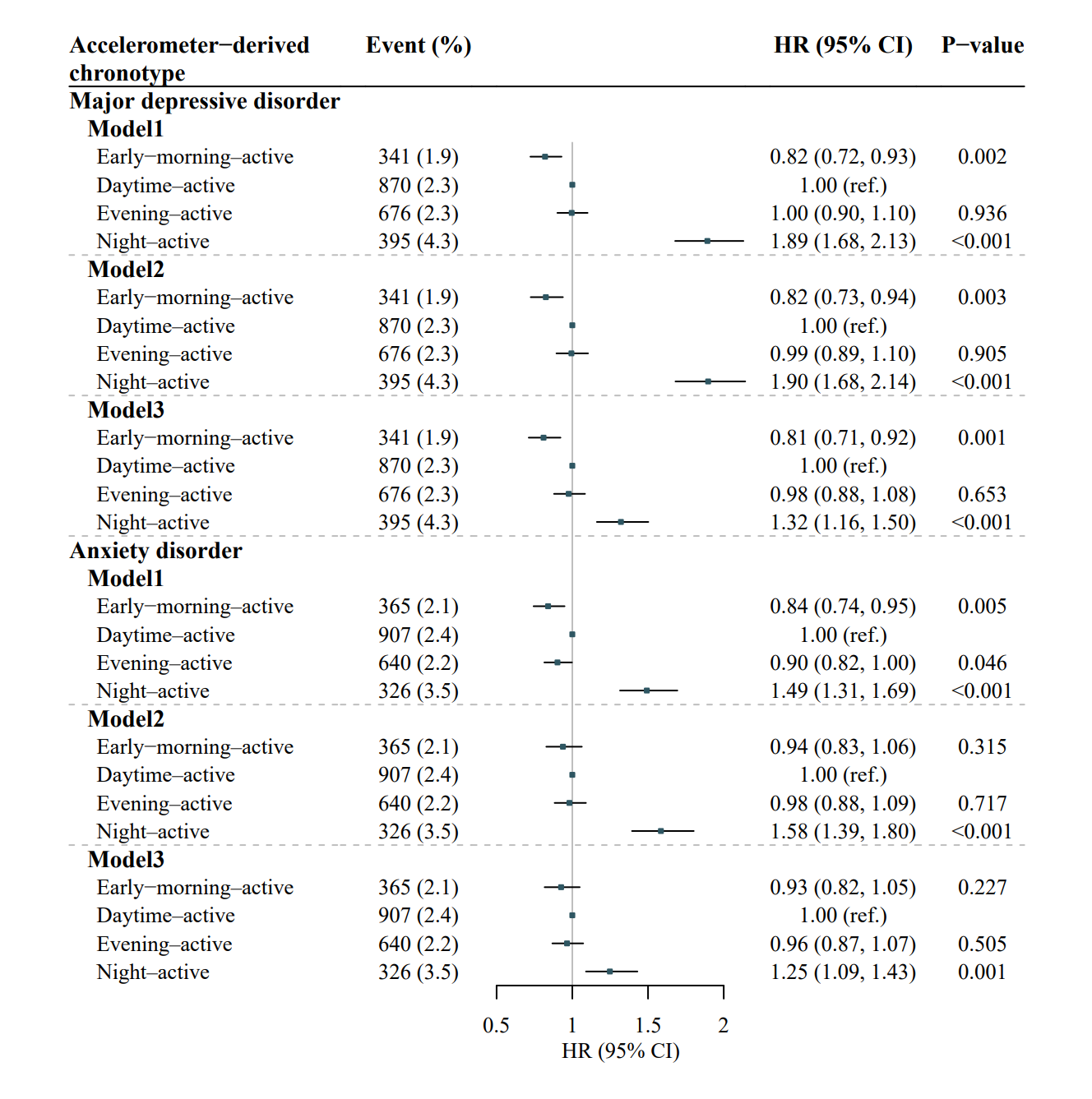


**Figure S12** Association of accelerometer-derived rest-activity timing phenotypes with mental disorders among participants without sleep disorders at baseline (N=93,340).

Model 1 (basic) was unadjusted model. Model 2 (sociodemographic) was adjusted for age at accelerometry test, gender, ethnicity, education level, quintiles of TDI. Model 3 (sociodemographic and lifestyle) was adjusted for smoking status, alcohol drinking frequency, diet score, BMI, sleep score, moderate-vigorous physical activity time, besides covariates in model 2


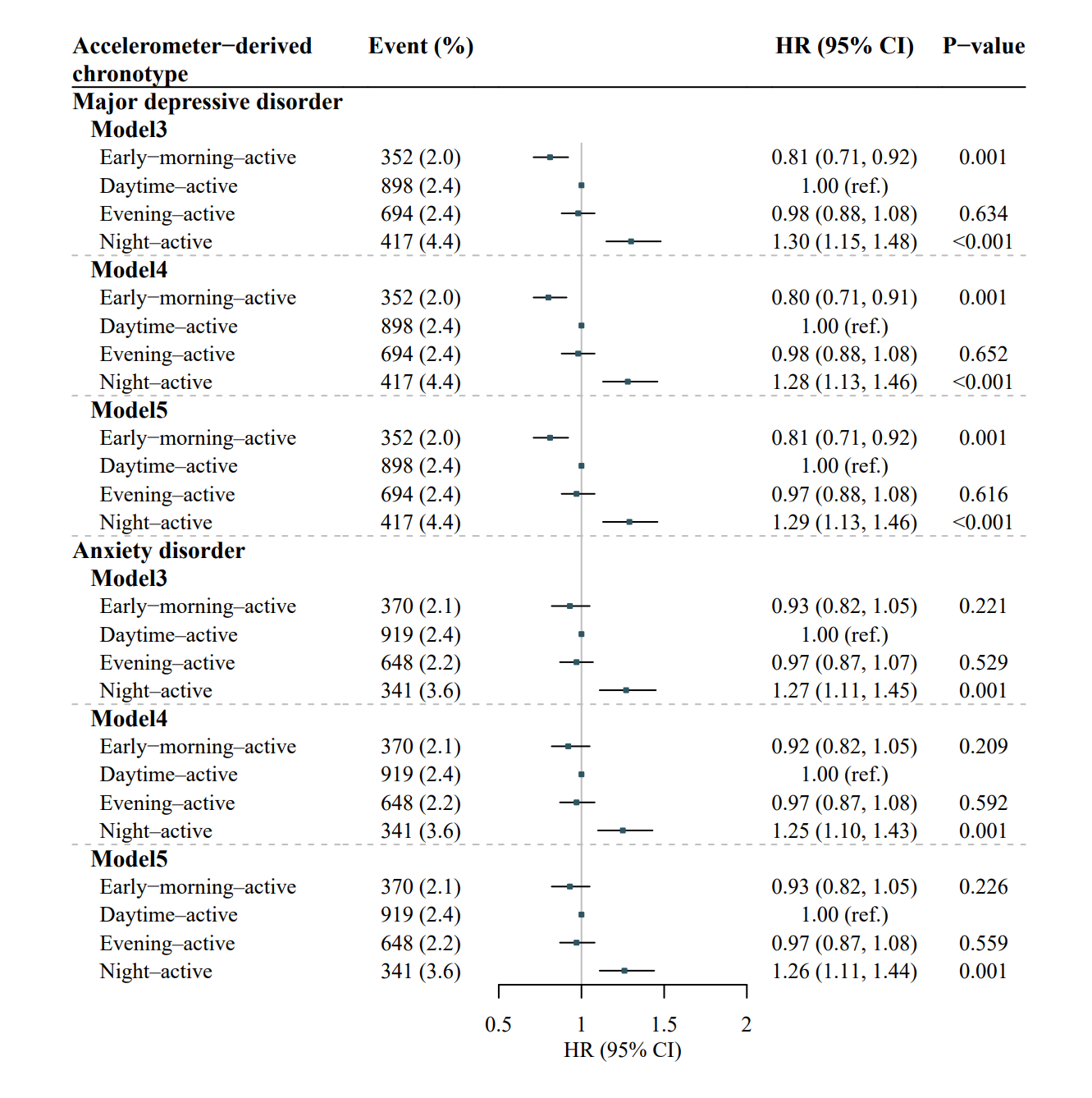


**Figure S13** Extended models additionally adjusting for prevalent chronic conditions (hypertension, diabetes, and cancer) and sleep disorders (N=94,344).

Model 3 was adjusted for age at accelerometry assessment, sex, ethnicity, educational level, and quintiles of the Townsend Deprivation Index (TDI)，smoking status, alcohol drinking frequency, diet score, body mass index (BMI), sleep score, and moderate-to-vigorous physical activity duration. Model4 was adjusted for history of diabetes, history of hypertension, and history of cancer, besides covariates in model 3. Model5 was adjusted for sleep disorders, besides covariates in model 3.


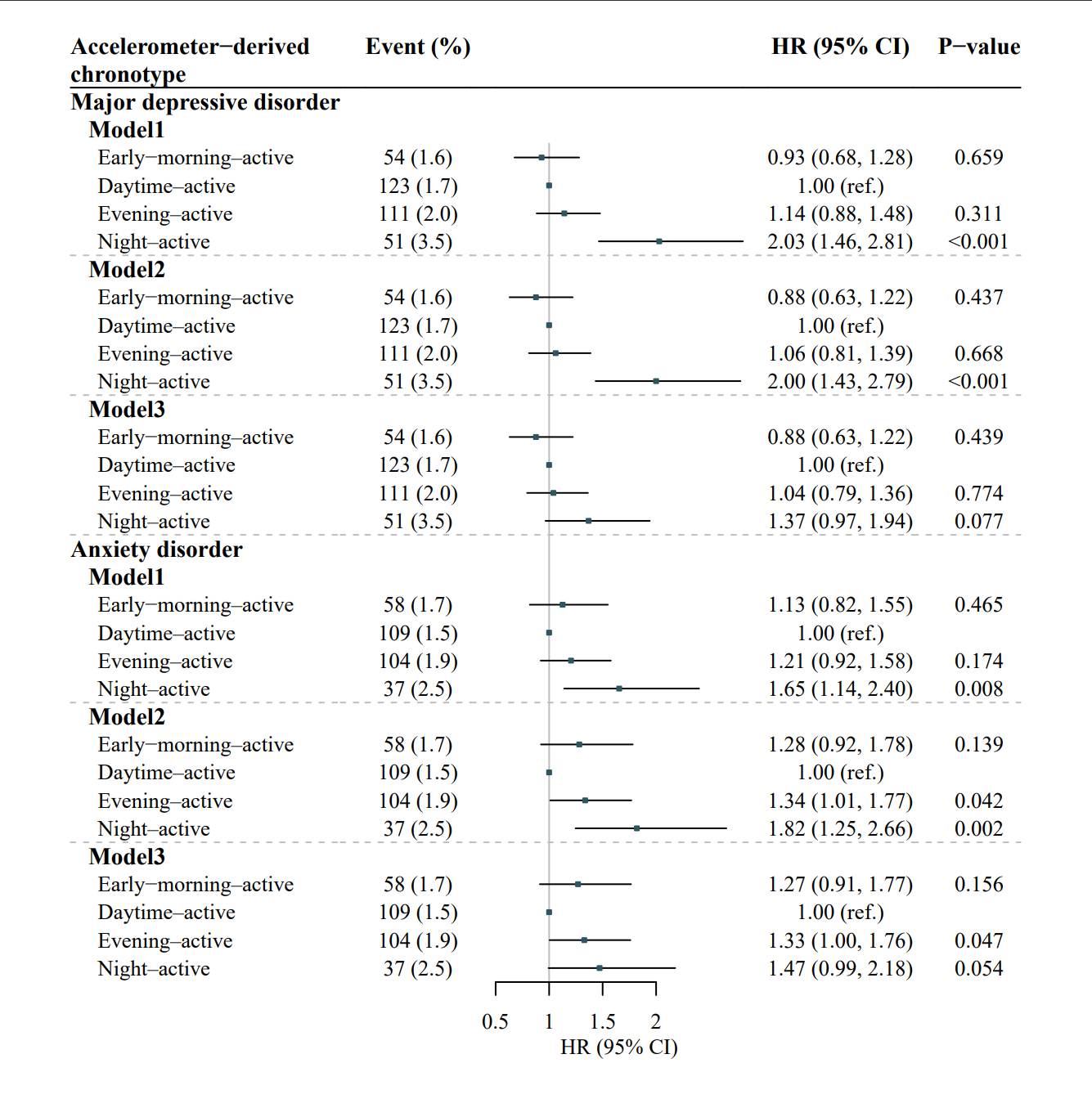


**Figure S14** Association of accelerometer-derived rest-activity timing phenotypes with mental disorders restricted to the MRI subcohort (N=17,571).

Model 1 (basic) was unadjusted model. Model 2 (sociodemographic) was adjusted for age at accelerometry test, gender, ethnicity, education level, quintiles of TDI. Model 3 (sociodemographic and lifestyle) was adjusted for smoking status, alcohol drinking frequency, diet score, BMI, sleep score, moderate-vigorous physical activity time, besides covariates in model 2.


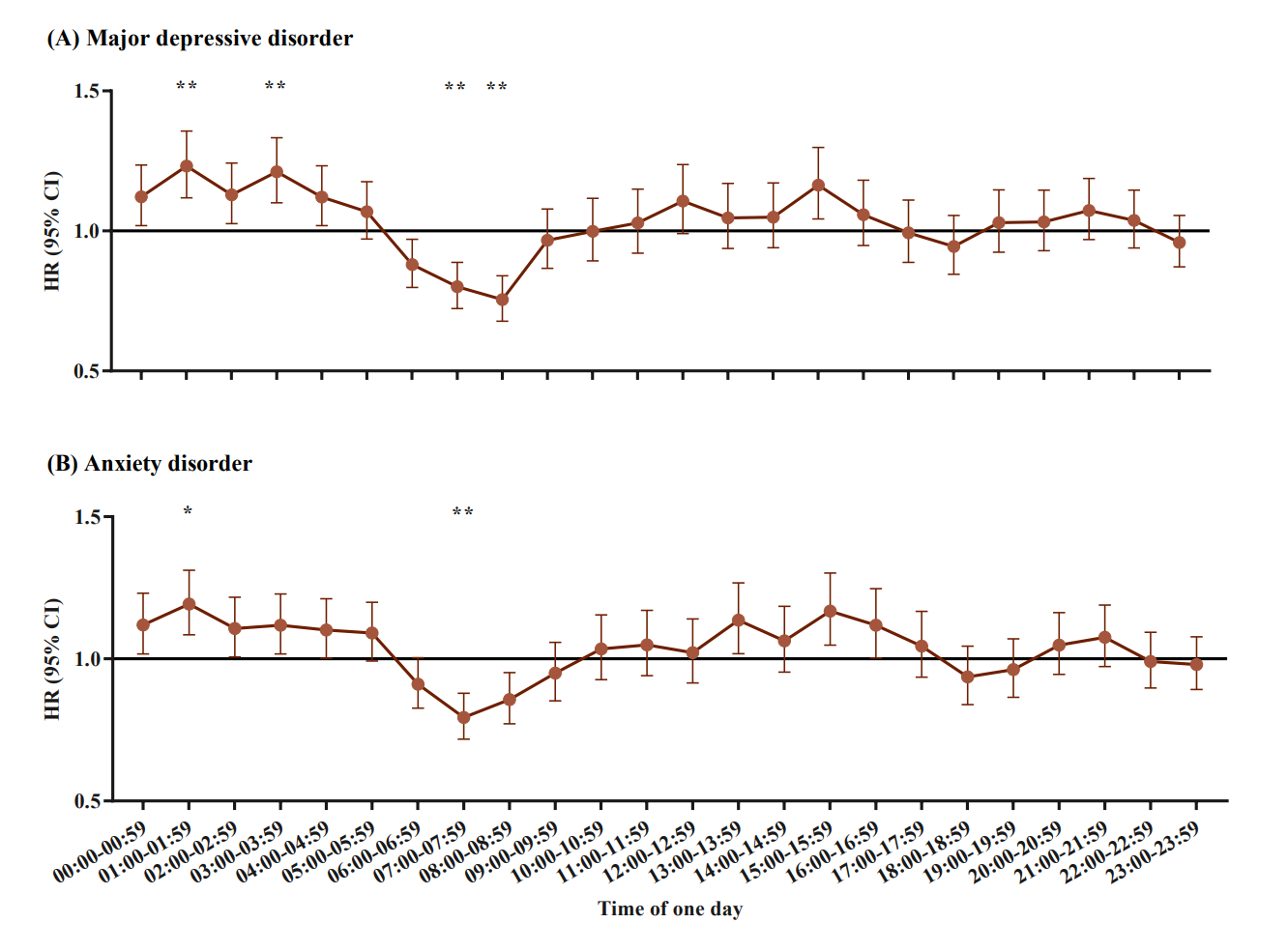


**Figure S15** Associations of PA intensity over the 24-hour cycle with mental disorders using 2-year landmark analysis (N=90,776).

Hazard ratios (HRs) and 95% confidence intervals (CIs) were from COX models comparing high and low PA levels (using the median as the cut-off point) of each one-hour period of 24-hour circle adjusted for age at accelerometry test, gender, ethnicity, education level, quintiles of TDI, smoking status, alcohol drinking frequency, diet score, BMI, sleep score, and moderate-vigorous physical activity time.

*P values were below the multi-comparisons threshold of 0.05 / 24 = 0.00208.

**P values were below the multi-comparisons threshold of 0.05 / (24*10) = 0.000208.

The cut-off points of each hour were listed in **Supplemental Table 2***.*

Abbreviations: PA, physical activity.

***
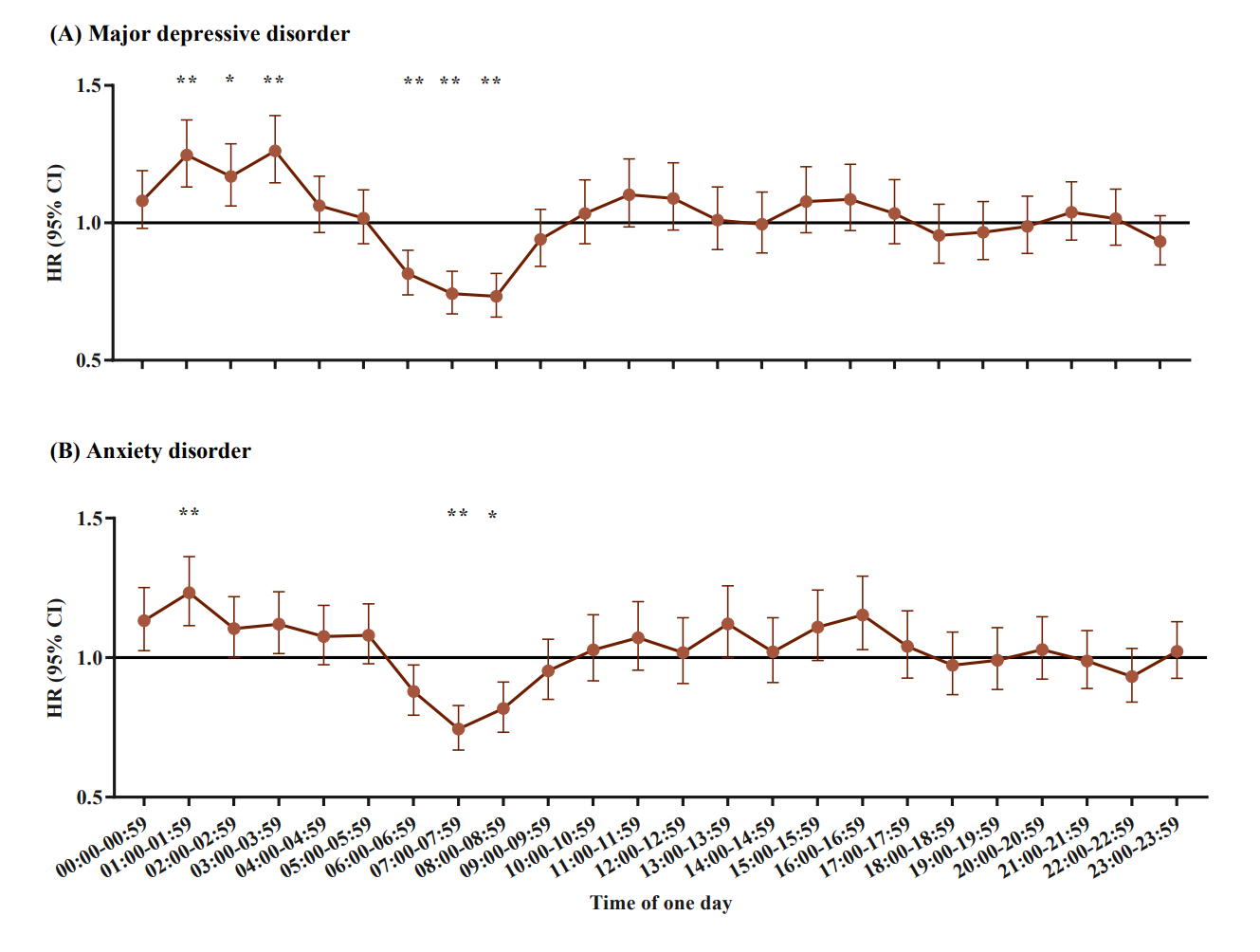
***

**Figure S16** Associations of PA intensity over the 24-hour cycle with mental disorders among participants with no missing value in covariates (N=71,853).

Hazard ratios (HRs) and 95% confidence intervals (CIs) were from COX models comparing high and low PA levels (using the median as the cut-off point) of each one-hour period of 24-hour circle adjusted for age at accelerometry test, gender, ethnicity, education level, quintiles of TDI, smoking status, alcohol drinking frequency, diet score, BMI, sleep score and moderate-vigorous physical activity time.

*P values were below the multi-comparisons threshold of 0.05 / 24 = 0.00208.

**P values were below the multi-comparisons threshold of 0.05 / (24*10) = 0.000208.

The cut-off points of each hour were listed in **Supplemental Table 2***.*

Abbreviations: PA, physical activity.

**Table S1** The description of physical activity intensity in each hour period over the 24-hour cycle.

| Hourly PA intensity | Median (mg) | P_25_ (mg) | P_75_ (mg) | Minim (mg) | Maxum (mg) |
| --- | --- | --- | --- | --- | --- |
| Average acceleration 00:00 - 00:59 | 4.45 | 2.92 | 8.64 | 0.39 | 1048.76 |
| Average acceleration 01:00 - 01:59 | 3.16 | 2.54 | 4.43 | 0.38 | 149.50 |
| Average acceleration 02:00 - 02:59 | 2.96 | 2.46 | 3.74 | 0.14 | 166.18 |
| Average acceleration 03:00 - 03:59 | 2.93 | 2.46 | 3.61 | 0.29 | 204.94 |
| Average acceleration 04:00 - 04:59 | 2.97 | 2.49 | 3.67 | 0.06 | 213.46 |
| Average acceleration 05:00 - 05:59 | 3.21 | 2.61 | 4.43 | 0.17 | 482.69 |
| Average acceleration 06:00 - 06:59 | 5.69 | 3.25 | 15.38 | 0.05 | 861.96 |
| Average acceleration 07:00 - 07:59 | 22.28 | 10.36 | 38.47 | 0.02 | 815.28 |
| Average acceleration 08:00 - 08:59 | 37.88 | 25.66 | 52.31 | 1.33 | 1015.24 |
| Average acceleration 09:00 - 09:59 | 43.94 | 32.49 | 57.92 | 0.87 | 1208.71 |
| Average acceleration 10:00 - 10:59 | 46.47 | 35.19 | 60.58 | 1.49 | 1108.20 |
| Average acceleration 11:00 - 11:59 | 45.89 | 35.07 | 59.19 | 0.57 | 829.89 |
| Average acceleration 12:00 - 12:59 | 44.95 | 35.07 | 57.01 | 1.27 | 946.07 |
| Average acceleration 13:00 - 13:59 | 41.70 | 32.42 | 53.24 | 1.57 | 759.53 |
| Average acceleration 14:00 - 14:59 | 40.81 | 31.11 | 52.97 | 2.09 | 509.41 |
| Average acceleration 15:00 - 15:59 | 40.06 | 30.16 | 52.15 | 2.10 | 659.26 |
| Average acceleration 16:00 - 16:59 | 38.53 | 28.87 | 50.36 | 2.13 | 597.32 |
| Average acceleration 17:00 - 17:59 | 37.70 | 28.08 | 49.28 | 1.60 | 702.53 |
| Average acceleration 18:00 - 18:59 | 35.55 | 26.35 | 46.93 | 2.10 | 665.80 |
| Average acceleration 19:00 - 19:59 | 30.21 | 21.78 | 40.97 | 1.88 | 685.28 |
| Average acceleration 20:00 - 20:59 | 23.01 | 16.25 | 32.34 | 1.96 | 472.25 |
| Average acceleration 21:00 - 21:59 | 18.62 | 13.11 | 26.23 | 1.58 | 510.13 |
| Average acceleration 22:00 - 22:59 | 18.81 | 13.33 | 25.81 | 1.30 | 406.26 |
| Average acceleration 23:00 - 23:59 | 11.50 | 6.32 | 18.31 | 0.81 | 709.86 |

**Table S2** Covariate definitions in multivariate regression models.

| **Covariates** | **Resources** | **Definitions** |
| --- | --- | --- |
| Smoking status | A touchscreen questionnaire at baseline assessment | Smoking status was derived from a touchscreen questionnaire. Current smokers were defined as smoking on most or all days or only occasionally according to their response to the question, “Do you smoke tobacco now?”. Non-smokers were defined as those who have never smoked according to their response to the question above and those who just tried once or twice according to their answer to “In the past, how often have you smoked tobacco?” |
| Measured minutes per week of MVPA | Wrist-worn triaxial accelerometers | Minutes per week (min/week) of moderate to vigorous (MVPA) were determined as the time spent >125 mg extrapolated from fraction of time spent over the total wear time. |
| The diet quality score | A touchscreen questionnaire (food frequency questionnaire) at baseline assessment | The diet quality score was adopted from previously published studies. It was a 0 to 5 scale calculated by scoring five items each as one point, including vegetable intake above or equal to the median (four tablespoons each day), fruits intake above or equal to the median (3 pieces/day), fish intake above or equal to the median (once a week), unprocessed red meat intake less than the median (once a week), processed meat intake less than the median (once a week). A higher score indicates a much healthier diet. We further defined a diet score category as high (4-5), medium (2-3), and low (0-1) based on the continuous sleep index. |
| The sleep quality score | A touchscreen questionnaire at baseline assessment | The sleep quality score was calculated using 5 sleep-related self-reported items. It was a 0 to 5 scale calculated by scoring five items each as one point, including no self-reported snoring, no frequent daytime sleepiness (“never/rarely” or “sometimes”), normal sleep duration (7-8 h/day), reported never or rarely having insomnia symptoms, and getting up easy in morning (“fairly easy” or “very easy”). A higher index indicating a general better sleep quality. We further defined a sleep index category as high (4-5), medium (2-3), and low (0-1) based on the continuous sleep index. |

**Table S3** Tract loadings on the first principal component of white matter fractional anisotropy (gFA) and mean diffusivity (gMD)

| Field ID | Field | gFA | gMD |
| --- | --- | --- | --- |
| 25501 | Inferior fronto-occipital fasciculus (right) | 0.877 | 0.913 |
| 25503 | Inferior longitudinal fasciculus (right) | 0.874 | 0.893 |
| 25500 | Inferior fronto-occipital fasciculus (left) | 0.857 | 0.902 |
| 25510 | Superior longitudinal fasciculus (right) | 0.849 | 0.901 |
| 25502 | Inferior longitudinal fasciculus (left) | 0.842 | 0.875 |
| 25509 | Superior longitudinal fasciculus (left) | 0.833 | 0.901 |
| 25499 | Forceps minor | 0.815 | 0.792 |
| 25491 | Anterior thalamic radiation (right) | 0.804 | 0.832 |
| 25490 | Anterior thalamic radiation (left) | 0.798 | 0.836 |
| 25508 | Posterior thalamic radiation (right) | 0.735 | 0.740 |
| 25507 | Posterior thalamic radiation (left) | 0.699 | 0.692 |
| 25512 | Superior thalamic radiation (right) | 0.679 | 0.822 |
| 25511 | Superior thalamic radiation (left) | 0.675 | 0.830 |
| 25514 | Uncinate fasciculus (right) | 0.672 | 0.764 |
| 25513 | Uncinate fasciculus (left) | 0.653 | 0.710 |
| 25488 | Acoustic radiation (left) | 0.595 | 0.491 |
| 25489 | Acoustic radiation (right) | 0.593 | 0.543 |
| 25496 | Corticospinal tract (left) | 0.587 | 0.634 |
| 25497 | Corticospinal tract (right) | 0.584 | 0.647 |
| 25498 | Forceps major | 0.534 | 0.431 |
| 25492 | Cingulate gyrus part of cingulum (left) | 0.506 | 0.717 |
| 25493 | Cingulate gyrus part of cingulum (right) | 0.483 | 0.724 |
| 25504 | Middle cerebellar peduncle | 0.358 | 0.256 |
| 25494 | Parahippocampal part of cingulum (left) | 0.346 | 0.361 |
| 25495 | Parahippocampal part of cingulum (right) | 0.300 | 0.362 |
| 25506 | Medial lemniscus (right) | 0.261 | 0.165 |
| 25505 | Medial lemniscus (left) | 0.238 | 0.184 |
|  | Kaiser-Meyer-Olkin factor adequacy (KMO) | 0.93 | 0.94 |
|  | P-value of Bartlett's test | <0.001 | <0.001 |
|  | Cumulative contribution of variance | 0.437 | 0.493 |
|  | The root mean square of the residuals (RMSR) | 0.09 | 0.08 |

Standardized loadings are provided. Bilateral measures were introduced separately.

**Table S4** Baseline characteristics of participants with vs without MRI data

| Characteristics | All participants  (n=94,344) | MRI unavailable (n=76,773) | MRI available (n=17,571) | *P* | *SMD* |
| --- | --- | --- | --- | --- | --- |
| Age at accelerometry test, years (Mean (SD)) | 62.32 (7.9) | 62.48 (7.90) | 61.64 (7.60) | <0.001 | 0.109 |
| Male (No (%)) | 41459 (43.9) | 33480 (43.6) | 7979 (45.4) | <0.001 | 0.036 |
| White (No (%)) | 91102 (96.9) | 74022 (96.8) | 17080 (97.4) | <0.001 | 0.039 |
| College or university degree (No (%)) | 40735 (47.5) | 32334 (46.6) | 8401 (51.5) | <0.001 | 0.102 |
| Townsend deprivation index at recruitment (Median [IQR]) | -2.45 [-3.82, -0.20] | -2.41 [-3.80, -0.13] | -2.63 [-3.90, -0.50] | <0.001 | 0.078 |
| Smoking status (No (%)) |  |  |  |  |  |
| Never | 53920 (57.3) | 43165 (56.4) | 10755 (61.3) | <0.001 | 0.110 |
| Previous | 33793 (35.9) | 27955 (36.5) | 5838 (33.3) |  |  |
| Current | 6383 ( 6.8) | 5445 ( 7.1) | 938 ( 5.4) |  |  |
| Alcohol drinking frequency (No (%)) |  |  |  |  |  |
| At least three times a week | 46297 (49.1) | 37376 (48.7) | 8921 (50.8) | <0.001 | 0.076 |
| Less than three times a week | 33949 (36.0) | 27544 (35.9) | 6405 (36.5) |  |  |
| Special occasions only or never | 14023 (14.9) | 11785 (15.4) | 2238 (12.7) |  |  |
| Diet quality score (No (%)) |  |  |  |  |  |
| Low (0-1) | 9443 (10.0) | 7669 (10.0) | 1774 (10.1) | 0.909 | 0.004 |
| Medium (2-3) | 48695 (51.6) | 39640 (51.6) | 9055 (51.5) |  |  |
| High (4-5) | 36206 (38.4) | 29464 (38.4) | 6742 (38.4) |  |  |
| Sleep quality score (No (%)) |  |  |  |  |  |
| Low (0-1) | 43780 (49.5) | 35372 (49.2) | 8408 (50.7) | <0.001 | 0.036 |
| Medium (2-3) | 42741 (48.3) | 34893 (48.5) | 7848 (47.3) |  |  |
| High (4-5) | 1959 ( 2.2) | 1637 ( 2.3) | 322 ( 1.9) |  |  |
| Body mass index, kg/m^2^ (Mean (SD)) | 26.67 (4.49) | 26.73 (4.55) | 26.39 (4.21) | <0.001 | 0.080 |
| Overall average acceleration (no-wear time bias adjusted), mg, (Median [IQR]) | 27.25 [22.59, 32.71] | 27.12 [22.46, 32.59] | 27.78 [23.23, 33.17] | <0.001 | 0.090 |
| Moderate-vigorous physical activity, minutes/week, (Median [IQR]) | 453.60 [310.69, 628.69] | 449.06 [303.91, 624.96] | 473.76 [332.99, 650.07] | <0.001 | 0.107 |
| Average acceleration per hour for four periods of one day, mg, (Median [IQR]) |  |  |  |  |  |
| Morning (6:00-7:59) | 15.07 [7.32, 27.58] | 14.97 [7.28, 27.46] | 15.46 [7.52, 28.11] | 0.001 | 0.022 |
| Daytime (8:00-19:59) | 42.05 [34.44, 50.93] | 41.84 [34.22, 50.73] | 42.95 [35.47, 51.67] | <0.001 | 0.097 |
| Evening (20:00-00:59) | 16.15 [12.26, 21.36] | 16.08 [12.18, 21.31] | 16.49 [12.65, 21.62] | <0.001 | 0.049 |
| Night (01:00-05:59) | 3.27 [2.77, 4.20] | 3.27 [2.77, 4.22] | 3.23 [2.75, 4.11] | <0.001 | 0.021 |

Continuous variables were reported as mean (SD) or, for prespecified non‑normally distributed measures, as median (IQR). Normally distributed continuous variables included age at accelerometry assessment and body mass index, which were compared between groups using independent samples t-tests; non-normally distributed measures (Townsend deprivation index, accelerometer-derived minutes per week of moderate-to-vigorous physical activity, and accelerometer-derived rest-activity timing phenotype metrics) were analyzed with the Mann-Whitney U test. Categorical variables (sex, ethnicity, education, smoking status, alcohol drinking frequency, diet quality score, sleep quality score, and self-reported chronotype) were presented as counts (percentages), and intergroup comparisons were conducted using the chi-square test, with Fisher’s exact test applied if the expected frequency in any cell was < 5. Between‑group imbalance was quantified using standardized mean differences (SMD) rather than sole reliance on hypothesis test p-values.

**Table S5** Definitions of comorbidities and shift-work variables

| **Variable** | **Resources** | **UKB field ID(s) and coding** |
| --- | --- | --- |
| Bipolar disorder | Hospital inpatient records | ICD-10 F30-F31 |
| Psychotic disorders | Hospital inpatient records | ICD-10 F20-F29 |
| Attention deficit hyperactivity disorder | Hospital inpatient records | ICD-10 F90 |
| Substance use disorders | Hospital inpatient records | ICD-10 F10-F19, except for F10, F17 |
| Sleep disorders | Self-reported questionnaires at baseline assessment and hospital inpatient records | ICD-10 F51;  UKB Field ID 1123 |
| Cancer | Hospital inpatient records | ICD-10 C00-C99, except for C44 |
| Diabetes mellitus | Self-reported questionnaires at baseline assessment and hospital inpatient records | ICD-10 E10-14;  UKB Field ID 1220, 1222, 1223 |
| Hypertension | Self-reported questionnaires at baseline assessment and hospital inpatient records | ICD-10 I10-I13, I15;  UKB Field ID 1065, 1072 |
| Shift work or even night shift work* | A touchscreen questionnaire at baseline assessment | UKB Field ID 826, 3426 |

* Shift work and night shift status were assessed using two distinct self-reported questions, each with four response options (Never/rarely, Sometimes, Usually, Always):“Does your work involve shift work?”“Does your work involve night shifts?” Participants were categorized as having shift work or night shift exposure if they selected Sometimes, Usually, or Always for either (or both) questions. Those who responded Never/rarely to both questions were classified as the non-shift work group.
